# Antitrypanosomal and Antileishmanial Activities of *Tacca leontopetaloides* Tubers and *Zanthoxylum zanthoxyloides* Stem Bark

**DOI:** 10.3390/molecules30112468

**Published:** 2025-06-05

**Authors:** Elizabeth O. Agbo, John V. Anyam, Cyprian T. Agber, Christie A. Adah, Christopher Agbo, Augustina O. Ijeomah, Terrumun A. Tor-Anyiin, Hamed E. Alkhalaf, Aditya Sarode, Jamal I. Asseri, Alexander I. Gray, John O. Igoli, Harry P. De Koning

**Affiliations:** 1 Phytochemistry Research Group, Department of Chemistry, Joseph Sarwuan Tarka University, Makurdi PMB 2373, Benue State, Nigeria; lizzyagbo20885@gmail.com (E.O.A.); johnversh@gmail.com (J.V.A.); agbercyprian@gmail.com (C.T.A.); christieadah5@gmail.com (C.A.A.); 2 Department of Chemistry, Benue State University, Makurdi PMB 102119, Benue State, Nigeria; 3 Centre for African Medicinal Plant Research, North-Eastern University, Gombe 771104, Gombe State, Nigeria; 4 Department of Microbiology, Joseph Sarwuan Tarka University, Makurdi PMB 2373, Benue State, Nigeria; agbochristopher03@gmail.com; 5 Department of Chemistry, Joseph Sarwuan Tarka University, Makurdi PMB 2373, Benue State, Nigeria; tinaijeomah@gmail.com (A.O.I.); toranyiint@yahoo.com (T.A.T.-A.); 6 School of Infection and Immunity, College of Medical, Veterinary and Life Sciences, University of Glasgow, Glasgow G12 8TA, UK; 2932662a@student.gla.ac.uk (H.E.A.); 2264761a@student.gla.ac.uk (J.I.A.); 7 Department of Pure and Applied Chemistry, University of Strathclyde, Glasgow G4 0RE, UK; aditya.sarode@strath.ac.uk; 8 Strathclyde Institute of Pharmacy and Biomedical Science, University of Strathclyde, Glasgow G4 0RE, UK; a.i.gray@strath.ac.uk

**Keywords:** *Tacca leontopetaloides*, tarkalynin A, tarkalynin B, taccalonolides, antitrypanosomal, antileishmanial activity, *Zanthoxylum zanthoxyloides*, dihydrochelerythrin, fagaramide

## Abstract

The phytochemical screening of extracts of *Tacca leontopetaloides* tubers has afforded the isolation of two novel chalcones, tarkalynins A and B, along with taccalonolide A and its 12-propanoate. The screening of *Zanthoxylum zanthoxyloides* stem bark yielded taraxerol acetate, dihydrochelerythrin and fagaramide. These compounds were obtained through column and thin-layer chromatography and identified using NMR and LC-HRMS. The compounds were tested against *Trypanosoma brucei brucei* s427 and its multi-drug-resistant clone B48, against *Trypanosoma evansi*, *Trypanosoma equiperdum* and *Trypanosoma congolense*, and against *Leishmania mexicana*. Cytotoxicity was tested against the human HEK293 cell line. The highest activities were observed with dihydrochelerythrin and fagaramide against *T. b. brucei* s427 and B48, *T. evansi*, and *L. mexicana*, with EC_50_ values of 1.37, 2.559, 1.09, and 5.44 µM and 17.8, 10.9, 10.9, and 13.3 µM, respectively. In addition, tarkalynin A and taraxerol acetate displayed promising activity against *T. equiperdum* (EC_50_ = 21.4 and 21.3 µM, respectively). None of these compounds showed significant cross-resistance with existing trypanocides (RF ≈ 1; *p* > 0.05). The compounds displayed low toxicity to human cells, with most exhibiting no growth inhibition at concentrations of 100, or even 300 µM. This report provides further evidence of the potential use of natural products for combating parasitic diseases.

## 1. Introduction

African trypanosomes are the etiological agents of a wide range of diseases and are usually transmitted by insect vectors feeding on humans (Human African Trypanosomiasis or sleeping sickness) and animals (African Animal Trypanosomiasis or AAT) [1]. This mode of transmission is true for all pathogenic trypanosomes, except *Trypanosoma equiperdum*, which is transmitted sexually by copulation in horses and other equids, causing a wasting disease known as dourine [2]. Sleeping sickness, caused by *T. gambiense* and *T. rhodesiense*, is progressing towards control and elimination [3], but animal trypanosomiasis cases continue to grow in number and geographical scope [4,5,6]. AAT is commonly known as nagana in sub-Saharan Africa, where it is mostly transmitted by tsetse flies, and is classified as a neglected tropical veterinary disease. It is also referred to as surra in North Africa and Asia, mal de caderas and Derrengadera in South America, and as *dourine* for the sexually transmitted form in equines [7]. The main species responsible for nagana are *T. brucei brucei*, *T. congolense,* and *T. vivax*, whereas *T. evansi* causes surra and together with *T. vivax* contributes to mal de caderas in South America [6,7,8,9]. Mutations in *Trypanosoma* sp. have led to the emergence of different drug-resistant strains to the current drugs used at different treatment stages [10]. Moreover, most of the available drugs are only effective against the hemolymphatic stage of the disease, before the infection spreads to the central nervous system [11]. There is no effective chemotherapy for several of the animal trypanosomiasis infections, most notably dourine, but drug resistance threatens the treatability of surra and nagana as well [6,10,12]. This implies that AAT can no longer be effectively treated, and this situation is affecting livelihoods, economies, and food security [4,7].

Leishmaniasis, caused by protozoan parasites of up to 20 different *Leishmania* species, is also a public health and veterinary concern in many countries in the tropics and subtropics, with about one million new human cases annually [13]. It is transmitted through the bite of an infected female phlebotomine sandfly [14]. Current chemotherapies depend on treatment with drugs of various efficacies, availability, and toxicities, coupled with resistance as the greatest challenge [15].

Plants produce diverse active substances used in many fields of medicine, with proven templates for new drug development [11,16]. Tacca leontopetaloides, commonly referred to as Polynesian arrow or bat flower, Amura or Tarayaya (in Hausa), *Gbache* (in Tiv), or *Andru* (in Idoma) [17], is a perennial herbaceous plant commonly found in the North Central and Western parts of Nigeria [18]. It is a flowering plant with a highly bitter and starchy bulb-like tuber, which is used as a staple food and as a source of starch in North Central Nigeria [18,19,20]. Several compounds of pharmaceutical importance have been isolated from the plant with ethnopharmacological activities, including antimicrobial and antioxidant [21,22], anti-inflammatory and anti-pyretic [23,24], and anti-cancer properties [25]. Most of these compounds are taccalonolides, which belong to a class of highly oxygenated pentacyclic triterpenes. In cancer research, they have been shown to stabilize microtubules, similar to the anticancer agents paclitaxel and epothilone, and are active against cell lines resistant to those drugs [26]. The diversity in structure, unique mechanism of action, and low toxicity of taccalonolides have attracted interest for drug discovery. Consequently, several taccalonolides [20,26,27] have been isolated, as well as taccabulins A-E, evelynin A [28], and evelynin B [29]. The initial anti-protozoan report on taccalonolides [27] showed these compounds to possess a range of activities from promising to moderate (0.76 ≤ EC_50_ ≤ 12.2 µg/mL), and demonstrated their potential as drugs against *Trypanosoma* and *Leishmania* species.

We also assess here the anti-parasite properties of *Zanthoxylum zanthoxyloides*, popularly known as *Fagara*, *Candlewood*, *zanthoxylum*, and to some natives of Nigeria as *Akenaka* or *Ayer* (in Tiv), *Ufu otachacha* (in Igede), and *Faschuari* (in Hausa) [17]. It is a spiny and rather scandent shrub, up to 6–8 m tall, belonging to the family Rutaceae. It has been reported to contain α-pinene, citronellol, geraniol, limonene, β-myrcene [30], tannin, saponins, flavonoids, phenol, alkaloids, terpenoids, essential oils and coumarins, and to enact several medicinal activities including antinociceptive, antimalarial, cytotoxic, antiproliferative, anthelminthic, antiviral and antifungal, antioxidant, analgesic, anti-inflammatory, antimicrobial, wound-healing, larvicidal, trypanocidal, uterine contraction, antitumor and hepatoprotective properties [31,32,33,34]. Here, we report a further investigation of extracts from this plant to isolate compounds possessing higher activity against parasites causing HAT, AAT, or leishmaniasis, with possible different modes of action and no cross-resistance to existing treatments.

## 2. Results and Discussion

### 2.1. Isolation and Structural Characterization

The purification of compounds from the ethyl acetate extracts of *Tacca leontopetaloides* tubers afforded a novel chalcone, tarkalynin A (**1**), belonging to the taccabulin class of compounds. Three previously known compounds were also identified in the *T. leontopetaloides* extracts. First, a methylenedioxy dihydrochalcone (**2**) from the combined hexane and ethyl acetate extracts of the *Tacca* peels in fraction TPHE 26 was identified as 1-(benzo[d][1′,3′]-dioxol-5′-yl)-3-(2″-hydroxy-4″,6″-dimethoxyphenyl)propan-1-one (tarkalynin B), and we here report its isolation as a natural compound for the first time; it has previously only been produced synthetically, as an intermediate to the synthesis of some taccabulins [35]. Second, taccalonolide A (**3**) was obtained from the combined hexane and ethyl acetate fractions of the *Tacca* tubers (TTHE 70–76) and peels (TPHE 62–74), and from the methanol fractions of the peels (TPM 49–69). Thirdly, taccalonolide A 12-propanoate (**4**) was isolated from the tuber hexane and ethyl acetate fraction TTHE 73 and peel methanol extract fractions TPM 20–37. Taraxerol acetate (**5**) was obtained from the ethyl acetate fraction of *Zanthoxylum zanthoxyloides* (ZSE-06), dihydrochelerythrin (**6**) was isolated from the ethyl acetate fraction of *Z. zanthoxyloides* (ZSE-42), and fagaramide (**7**) from *Z. zanthoxyloides* stem bark hexane fraction ZSH-44.

**Figure 1:**
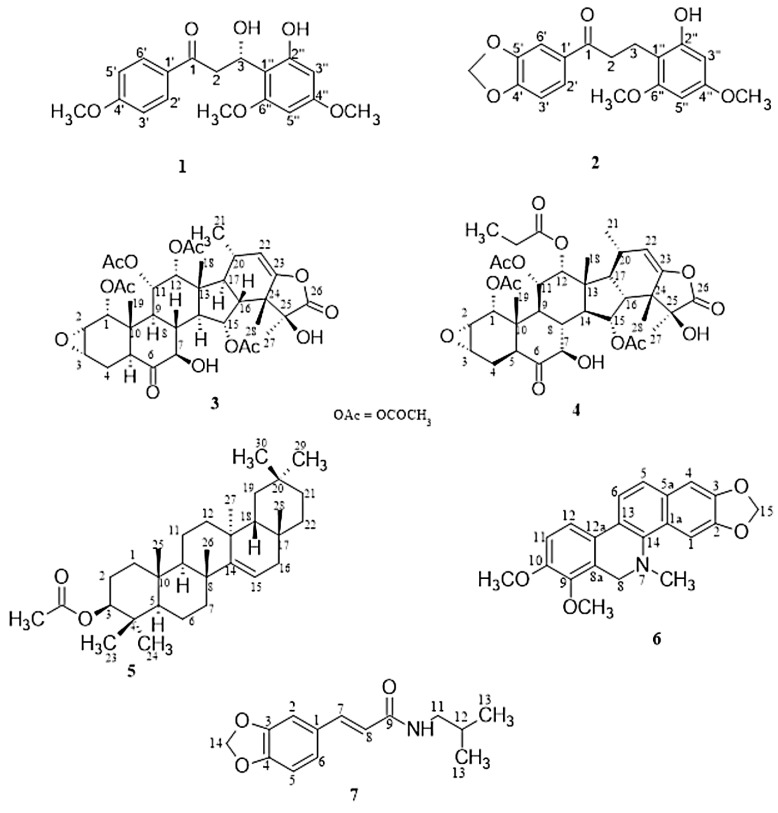
Structures of isolated compounds: **1**, tarkalynin A; **2**, tarkalynin B; **3**, taccalonolide A; **4**, taccalonolide A 12-propanoate; **5**, taraxerol acetate; **6**, dihydrochelerythrin; **7**, fagaramide.

Compound **1** was obtained from the ethyl acetate fractions (TTHE 48) of *Tacca* tubers as a brown solid. Its LC-HRMS spectrum yielded a molecular ion [M + H]^+^ at *m*/*z* 333.1336 (calculated 333.1338, C_18_H_21_O_6_), corresponding to the molecular formula C_18_H_20_O_6_, with seven degrees of unsaturation. This was confirmed by its ^1^H-NMR spectrum (Table 1), which showed four aromatic proton signals at *δ*_H_ (ppm) 8.09 (d, *J* = 9.0 Hz, H-2′ and 6′), 7.04 (d, *J* = 9.0 Hz, H-3′ and H-5′), 6.23 (d, *J* = 2.3 Hz, H-3″) and 6.05 (d, *J* = 2.3 Hz, H-5″) and a set of methylene signals at *δ*_H_ (ppm) 3.46 (dd, 14.9, 2.9, H-2a) and 2.86 (dd, 14.9, 7.6, H-2b). The integration of the proton spectrum showed that the signals at 7.04 and 8.09 ppm are due to two protons each, indicating symmetry in the aromatic ring. The spectrum also showed that the methylene protons are coupled to an oxymethine proton at 5.18 (dd, *J* = 7.6, 2.9, H-3). Three methoxy singlet signals were observed at *δ*_H_ (ppm) 3.95, 3.79, and 3.61. The ^13^C-NMR spectrum showed a total of eighteen signals including two quaternary aromatic carbons at *δ*_C_ (ppm) 125.9 (C-1′) and 105.1 (C-1″); four aromatic tertiary carbons at *δ*_C_ (ppm) 164.4 (C-4′), 160.4 (C-4″), 158.3 (C-6″) and 157.7 (C-2″); and six aromatic CH at 131.5 (C-2′ and C-6′), 114.0 (C-3′ and C-5′), 94.8 (C-3″) and 91.3 (C-5″) indicating the presence of a disubstituted and a tetrasubstituted aromatic ring. A characteristic signal at *δ*_C_ 197.9 ppm was indicative of an open-chain saturated ketone. The three signals at *δ*_C_ (ppm) 55.6, 55.3, and 55.4 confirmed the presence of three methoxy carbons substituted on the aromatic rings. Two aliphatic signals at *δ*_C_ (ppm) 75.6 and 30.5 were attributed to an oxygenated carbon and a methylene carbon, respectively. The structure was fully deduced using its 2D NMR (COSY, HSQC, HMBC) spectra. Its COSY spectrum showed correlations between the aromatic protons at 8.09 (H-2′ and H-6′) and 7.04 (H-3′ and H-5′), indicating they were ortho-coupled. There were also correlations between the oxymethine proton at 5.18 (H-3) and the methylene protons at 3.46 (H-2a) and 2.86 (H-2b). The HMBC spectrum showed long-range correlations between the aromatic protons at 7.04 (H-3′ and H-5′) and the quaternary carbon at 125.9 (C-1′), while the protons at 8.09 (H-2′ and H-6′) had correlations with the quaternary carbon at 164.4 (C-4′) and the carbonyl carbon at 197.9. Long-range correlations from the protons at 8.09 (H-2′ and H-6′) to the carbonyl carbon at 197.9, and from the methylene proton at 2.86 (H-2b) to two aromatic quaternary carbons at 105.1 (C-1″) and 157.7 (C-2″), confirmed the propanone chain to be substituted by aromatic rings at both ends. The three methoxy groups were identified by their HSQC correlations with their respective carbons: 3.95 (*δ*_C_ 55.6), 3.79 (*δ*_C_ 55.3), and 3.61 (*δ*_C_ 55.4). The methoxy-bearing carbons were identified by the HMBC correlations of the methoxy protons: 3.95 with *δ*_C_ 164.4, 3.79 with *δ*_C_ 160.4, and 3.61 with *δ*_C_ 158.3. Thus, compound **1** was characterized as (S)-3-hydroxy-3-(2″-hydroxy-4″,6″-dimethoxyphenyl)-1-(4′-methoxyphenyl)propan-1-one with a common name tarkalynin A (Figure 1); the yield was 14 mg, purity 85%. The stereochemistry of the 3-OH in structure 1 was determined from the coupling constants of H-3 (*J* = 7.6, 2.9 Hz) with the protons at C-2 (*J* = 14.9, 2.9 Hz, H-2a) and (*J* = 14.9, 7.6 Hz, H-2b). This is in contrast to the coupling constants reported for H-3 (*J* = 13.5, 2.7 Hz) by Xiao et al. [36]. Hence, the C-3 hydroxyl group in compound 1 must have the opposite conformation (lower wedge) to the stereochemistry (upper wedge) determined and reported for their 3-OH.

Compound **2** was isolated from the combined hexane and ethyl acetate extracts of the *T. leontopetaloides* peels in column fraction 26 (TPHE 26) as a brown solid (yield was 15 mg, purity 70%) with a molecular formula C_18_H_18_O_6_ from its LC-HRMS spectrum, which gave a molecular ion [M + H]^+^ at *m*/*z* = 331.1168 (calculated 331.1182, C_18_H_19_O_6_) with eight degrees of unsaturation. Its ^1^H-NMR spectrum gave signals (Table 1) for five aromatic protons at *δ*_H_ (ppm) 7.63 (dd, *J* = 8.3,1.8 Hz, H-2′), 7.47 (d, *J* = 1.8 Hz, H-6′), 6.84 (d, *J* = 8.3 Hz, H-3′), 6.20 (d, *J* = 1.8 Hz, H-3″) and 6.07 (d, *J* = 2.5 Hz, H-5″), indicating the compound has two aromatic rings and was similar to compound **1**. The only difference was a methylenedioxy group attached to one of the aromatic rings and the presence of neighboring methylene protons at 3.33 (H-2) and 2.95 (H-3), as indicated by the COSY correlations between them. From correlations in its 2D spectra, it was identified as 1-(benzo[d][1′,3′]-dioxol-5′-yl)-3-(2″-hydroxy-4″,6″-dimethoxyphenyl)propan-1-one and named tarkalynin B. The spectral data agree with the literature reports [35].

Compound **3** was isolated from the hexane and ethyl acetate extract fractions of the *T. leontopetaloides* tubers (TTHE 70–76) and peels (TPHE 62–74), and the methanol extract fractions of the peels (TPM 49–69) as a brown solid; yield = 206 mg, purity 95%. It was identified as taccalonolide A via the comparison of its spectral data with those reported in the literature [27]. Its LC-HRMS spectrum gave a molecular ion [M + H]^+^ at *m*/*z* 703.2973 (calculated 703.2966, C_36_H_47_O_14_), corresponding to the molecular formula C_36_H_46_O_14_.

Compound **4** was identified as Taccalonolide A 12-propanoate by comparison of its ^1^H-NMR data with literature reports [27]. It was also obtained as a brown solid (yield = 39 mg, purity 90%) from the hexane and ethyl acetate fractions of the *T. leontopetaloides* Tacca tubers (TTHE 73) and the peel methanol fractions (TPM 20–37). Its LC-HRMS spectrum yielded a molecular ion [M + H]^+^ at *m*/*z* 717.3156 (calculated 717.3122, C_37_H_49_O_14_), corresponding to the molecular formula C_37_H_48_O_14_.

Compound **5** was obtained from the ethyl acetate extract fraction ZSE-06 of *Z. zanthoxyloides* stem bark as a white solid (yield = 47 mg, purity 98%), and identified as taraxerol acetate by comparison of its ^1^H-NMR data with literature reports [37].

Compound **6** was also obtained from the ethyl acetate extract fraction ZSE-42 of *Z. zanthoxyloides* stem bark as a white solid (yield = 29 mg, purity 90%, and identified as dihydrochelerythrin by comparison of its spectral data with literature reports [38].

Compound **7** was obtained from the hexane extract fraction ZSH-44 of *Z. zanthoxyloides* stem bark as a white solid (yield = 24 mg, purity 90%) and identified as fagaramide by comparison of its spectral data with earlier reports [39].

### 2.2. Effect of the Isolated Compounds on Trypanosomes

The in vitro activities of six of the isolated compounds were assessed against bloodstream forms of *T. b. brucei* (s427 wild-type), multi-drug-resistant *T. b. brucei* (B48), *T. evansi* (WT) and *T. equiperdum* (WT), and *T. congolense* (WT) promastigotes, using a resazurin-based assay. All values are displayed in Table 2.

Dihydrochelerythrin (**6**) exhibited a very high activity, with EC_50_ values between 1.37 and 3.09 µM against all the trypanosomes of the *Trypanozoon* subgenus (i.e., *T. b. brucei*, *T. evansi* and *T. equiperdum*), including the multidrug-resistant clone B48 (*p* > 0.05 relative to s427). Against *T. congolense* (subgenus *Nannomonas*), the activity was significantly (*p* < 0.001) lower, although still promising with an EC_50_ value of 8.3 ± 0.6 µM. Since toxicity against HEK 293 was low, the in vitro selectivity index (SI) was good, especially for the *Trypanozoon* species (30.4 < SI < 87). Fagaramide (**7**) also displayed good activity against all trypanosome species (10.9 < EC_50_ < 34.4 µM), and was less selective for the *Trypanozoon* subgenus, as the small difference between *T. b. brucei* s427 and *T. congolense* was not statistically significant (*p* > 0.05). With toxicity against the human cell line above 364 µM, the SI values ranged from 10.6 to 33.8. This confirms a previous report by [39], who also reported an interestingly high antitrypanosomal activity of fagaramide against *T. b. brucei* s427, with EC_50_ = 7.3 µM and no toxicity to normal cell lines (macrophages RAW264.7; EC_50_ = 214.96 µM). This compares to the activity displayed by our compound **7** (fagaramide) with an EC_50_ of 17.8 µM against s427. *Z. zanthoxyloides* has been shown to possess anticancer [40,41] and antibacterial [32] properties.

Of the *T. leontopetaloides*-derived compounds, the novel compound Tarkalynin A (**1**) displayed the most promising activity, but again with a highly significant separation between the *Trypanozoon* and *Nannomonas* subgenera (*p* < 0.001), as EC_50_ values against the former ranged between 21.4 µM (*T. equiperdum*) and 46.0 µM for *T. evansi*, whereas the compound was almost inactive against *T. congolense* (EC_50_ = 246 µM). No EC_50_ could be determined against HEK 293 because it was non-toxic to these cells even at the highest concentration tested, 100 µg/mL (300 µM).

Taccalonolide A (**3**) and its 12-propanoate (**4**) displayed highly similar but moderate activities against the various trypanosome species. The small change from acetate to propanoate did not significantly impact its anti-trypanosomal properties. Although the compounds were not toxic to HEK 293 cells under the limited tested, their antiparasite activity was not sufficiently promising for them to be considered as lead compounds. Similarly, Taraxerol acetate (**5**) performed relatively poorly against most trypanosome species (EC_50_ > 60 µM), with only the EC_50_ for *T. equiperdum* lower, at 21.3 µM. Compounds **4** and **5** were not toxic to HEK 293 cells at the highest concentrations tested (100 and 200 µg/mL, respectively (140 µM, 427 µM)).

Cross-resistance with existing trypanocides of the diamidine class (e.g., pentamidine, diminazene, furamidine) and melaminophenyl arsenical class (melarsoprol, cymelarsan) was tested by a side-by-side comparison of the standard drug-sensitive strain *T. b. brucei* s427 and clone B48. This clone was derived from s427 by sequentially deleting the TbAT1 drug transporter [42] and in vitro exposure to pentamidine [43]; it is highly resistant to all these important drugs against HAT and AAT [43,44]. None of the compounds here tested exhibited significant resistance in B48, nor as much as a 2-fold higher EC_50_, while resistance to pentamidine was approximately 50-fold (*p* < 0.001). The diamidine and arsenical resistance in *T. brucei*, *T. evansi* and *T. equiperdum* is associated with the functional loss of two drug transporters: the aminopurine transporter P2/TbAT1 and the aquaporin TbAQP2 [10,45,46,47], as has been demonstrated for B48 [47]. Thus, the trypanocidal action of the compounds used in this study is not dependent on these crucial, common drug transporters.

In general, the compounds displayed lower activities against *T. congolense* than against the *Trypanozoon* subgenus trypanosome species. This is also observed with several important trypanocides, such as diminazene, pentamidine, other mitochondrion-targeting drugs, and the arsenicals [12,48,49]. This is a problem for the treatment of nagana, since *T. brucei* and *T. congolense* incidences almost completely overlap geographically, as they are transmitted by the same vectors, and therefore, the infecting species of a particular animal is rarely known [6,11]. However, dourine, in horses and other equids, is invariably caused by *T. equiperdum* [2], and surra, from North Africa to South Asia and the Philippines, is caused by *T. evansi* only [7,50]. In this context, it is important that **6** and **7** displayed the highest activities against *T. evansi* and almost as promising activity against *T. equiperdum*, and that new treatments for dourine and surra are urgently required.

The promising antitrypanosomal ability of dihydrochelerythrin and fagaramide may be due to the presence of the methylene-dioxy moiety. A report by Eze et al. [41] found that a derivative of dihydrochelerythrin, 6-Acetonyl-5, 6-dihydrochelerythrine, isolated from *Zanthoxylum leprieurii* showed no significant activity against *T. b. brucei* s427, and the structure–activity relationships of **6** should therefore be carefully studied.

There are no anti-protozoal activity reports for compounds obtained from *Tacca leontopetaloides,* except for our initial report on the anti-trypanosomal activity of compounds and fractions derived from the plant [27], wherein an activity of 3.13 µg/mL (4.6 µM) was displayed by taccalonolide A 12 propanoate and 11.42 µg/mL (16.3 µM) by taccalonolide A against *T. b. brucei* s427. Although taccalonolide A showed a moderate EC_50_ (45.4–66.3 µM), no cross-resistance (RF ≤ 1) was observed with the multi-drug-resistant B48, *T. evansi* and *T. equiperdum*. Taccalonolide A 12 propanoate (**4**) showed the least activity, but exhibited moderate activity against s427 with an EC_50_ of 53.4 µM and poor activity against B48, *T. evansi* and *T. equiperdum* in the range 75.1–52.7 µM. Generally, the tested compounds were poorly active against *T. congolense* (EC_50_ > 100 µM, except **7**—34.4 µM).

### 2.3. Effect of the Isolated Compounds on Leishmania mexicana

Dihydrochelerythrin (**6**) and fagaramide (**7**) showed significant activity (EC_50_ = 5.4 ± 0.6 µM and 13.3 ± 0.4 µM, respectively) against *L. mexicana*, and thus showed genuine and broad anti-kinetoplastid activity. Tarkalynin A (**1**) showed poor activity (EC_50_ = 581 µM) against *L. mexicana*. Neither EC_50_ nor SI values for taccalonolide A or its propanoate against *L. mexicana* could be obtained due to low activity.

### 2.4. Effect of the Isolated Compounds on HEK

Most of the compounds showed little to no toxicity against mammalian (HEK) cells at the highest tested concentration of 100 or 200 µg/mL, except for dihydrochelerythrin, which showed a moderate toxicity, with EC_50_ = 94.4 ± 23.2 µM.

## 3. Materials and Methods

### 3.1. Plant Collection

The tubers of *T. leontopetaloides* were collected from Kusuv (Buruku LGA, Benue State), and the stem bark of *Z. zanthoxyloides* from Bunu Tai (Tai LGA, Rivers State) in Nigeria. The plants were identified by the Forestry and Wildlife Department, Joseph Sarwuan Tarka University, Makurdi, Benue State, and the Department of Forestry and Environmental Studies, Rivers State University, Port Harcourt, with voucher specimen numbers UAM/FH/0469 and BSU/2017/ZZ-56. The tubers were rinsed with water, and the bark was scraped off, air-dried, ground, and sieved, while the stem bark was air-dried and ground into powder.

### 3.2. Extraction and Isolation of Constituents

The dried and ground tubers (6.9 kg) and peel (4.0 kg) of *T. leontopetaloides* and 1 kg stem bark of *Z. zanthoxyloides* were each macerated with hexane, ethyl acetate, and methanol (2500 mL, 48 h) successively. The filtrates were concentrated on a rotary evaporator at 40 °C and air-dried to obtain the hexane, ethyl acetate, and methanol extracts. These extracts were subjected to TLC on pre-coated silica gel plates and visualized using anisaldehyde-H_2_SO_4_ reagent. The hexane and ethyl acetate extracts of *T. leontopetaloides* showed similar profiles on TLC, and were therefore combined. Thus, four extracts were obtained: *Tacca* tuber hexane and ethyl acetate (TTHE), *Tacca* tuber methanol (TTM), *Tacca* peel hexane ethyl acetate (TPHE), and *Tacca* peel methanol (TPM). The maceration of *Z. zanthoxyloides* stem bark yielded three extracts: *Zanthoxylum* stem bark hexane (ZSH), *Zanthoxylum* stem bark ethyl acetate (ZSE), and *Zanthoxylum* stem bark methanol (ZSM). The extracts were subjected to column chromatography over silica gel 60 (0.063–0.200 mm for CC, Merck, Darmstadt, Germany). Each extract was pre-adsorbed onto silica gel and concentrated, in vacuo, to a free-flowing powder, loaded onto a wet-packed silica gel (150 g) column, and eluted gradient-wise using ethyl acetate in hexane, in 200 mL stepwise increases of 5% until 100% ethyl acetate for hexane and ethyl acetate extracts, while methanol in ethyl acetate was used similarly, but with 1% stepwise increases in methanol up to 10% methanol. The fractions (20 mL each) and numbers of vials collected were as follows: TTM 164, TPM 169, ZSM 100, TTHE 168, TPHE 162, ZSH 120, ZSE 150. Fractions with similar constituents were combined based on their TLC profiles.

### 3.3. Spectroscopic Analysis

The spectral analysis was carried out at the Institute of Organic Chemistry, University of Glasgow, Scotland. One- and two-dimensional NMR spectra of the compounds were obtained on a Bruker AVIII (400 MHz) spectrophotometer using deuterated chloroform (CDCl_3_) or acetone ((CD_3_)_2_CO) as solvents. The spectra were processed using MestReNova (Mestrelab Research, Santiago de Compostela, Spain), and chemical shifts were referenced against residual solvent peaks. Mass spectra were acquired on a QTOF high-resolution Agilent 6545 mass spectrometer (Agilent Technologies, Santa Clara, CA, USA) coupled to an Agilent Infinity 1290 UHPLC system (Agilent Technologies, Santa Clara, CA, USA).

### 3.4. Parasite Culture

In vitro cultures of *T. b. brucei*, *T. evansi* and *T. equiperdum*—Bloodstream trypomastigotes of *T. b. brucei* (s427 wild-type) [51], multi-drug-resistant *T. b. brucei* (B48) [43], *T. evansi* (AnTat 3/3) [52] and *T. equiperdum* (BoTat1) [46] were cultured in HMI-9 medium (Invitrogen, Paisley, UK) supplemented with 3.0 g/L NaHCO_3_, 14.3 µL/L β-mercaptoethanol adjusted to pH 7.4 and 10% (*v*/*v*) heat-inactivated Fetal Bovine Serum (FBS; Gibco Life Technologies, Paisely, UK), and incubated at 37 °C in a 5% CO_2_ atmosphere as described. The cells were passaged every 72 h.

In vitro cultures of *T. congolense*—Bloodstream forms of *Trypanosoma congolense* (IL3000, Wellman, Oldbury, UK) were cultured as described [53] in Tc-BSF3 medium at 34 °C in a 5% CO_2_ atmosphere. The basal medium (1 L) was prepared using 9.60 g MEM (Sigma M0643, Sigma-Aldrich, Gillingham, UK), 5.96 g HEPES, 2.20 g NaHCO_3_, 1 g D-glucose, 110 mg sodium pyruvate (Sigma P3662, Sigma-Aldrich, Gillingham, UK), 10.68 mg adenosine, 14 mg hypoxanthine, 4 mg thymidine and 14.10 mg bathocuproinesulfonic acid (Sigma B1125, Sigma-Aldrich, Gillingham, UK). The basal medium (150 mL) was then supplemented with 40 mL heat-inactivated fresh goat serum (GIBCO, Paisely, UK), 10 mL heat-inactivated serum plus (Sigma-Aldrich, Gillingham, UK), 2.8 µL β-mercaptoethanol, 1.6 mL glutamine and 2 mL penicillin/streptomycin solution to obtain 200 mL of Tc-BSF-3 medium [54]. The cells were passaged every 72 h.

In vitro *Cultures of L. Mexicana—Leishmania mexicana* promastigotes of strain MNY/BZ/62/M379 [55] were cultured in HOMEM medium supplemented with 10% heat-inactivated FBS and 1% penicillin/streptomycin solution (Gibco Life Technologies, Paisely, UK) at 27 °C as described [56]. The cells were passaged every 72 h.

In vitro cultures of HEK 293 cells—The Human Embryonic Kidney cell line HEK 293 strain was grown in a standard medium containing 500 mL Dulbecco’s modified Eagle’s medium (DMEM) (Sigma), 50 mL of heat-inactivated FBS (GIBCO, Paisely, UK), and 5 mL penicillin/streptomycin solution [57]. All constituents were mixed under sterile conditions in the DMEM bottle and stored at 4 °C before use. The cells were incubated at 37 °C and 5% CO_2_ and passaged twice a week in a vented flask until 80–85% confluence.

### 3.5. Antitrypanosomal and Antileishmanial Assays

The in vitro drug sensitivity assay in bloodstream forms of trypanosomes and *Leishmania*, which also assessed for toxicity to mammalian cells, was carried out at the School of Infection and Immunity, College of Medical, Veterinary and Life Sciences, University of Glasgow, Scotland, according to the Resazurin (Alamar blue^®^, Bio-Rad, Hercules, CA, USA) assay methods used in recent reports [48,56,58]. The assay is based on the reduction of the blue non-fluorescent dye resazurin sodium salt (Sigma) by living but not dead cells to the red fluorescent metabolite resorufin [58]. Stock solutions of isolated compounds were prepared at 10 mg/mL in dimethyl sulfoxide (DMSO (Merck, Darmstadt, Germany)), from which stocks of 200 μg/mL (400 μg/mL for *L. mexicana*) were prepared in the appropriate medium for each strain according to the standard protocol [59]. Exactly 200 μL of each stock was added to the first well of each row on a 96-well microplate, setting up for doubling dilutions of seven different drugs per plate, each over one row of a 96-well plate. Similarly, 200 μL of current trypanocides (positive control: diminazene aceturate for *T. congolense*, pentamidine for all other species) prepared at appropriate concentrations for each cell was added to the first well and included in each of the triplicate experiments. Next, 100 μL medium was pipetted using a multichannel micropipette into all remaining wells, and 100 μL of the drug was taken by a multichannel pipette from the first column and mixed gently with the medium in the wells of the second column, then another 100 μL from the second column well was added to the third column well and so on to the last-but-one column, creating a doubling dilution of 11 points. The last column of the plate was the drug-free negative control. Cell counts were performed using a hemocytometer, and cell density was adjusted to the desired concentration of cells/mL (2 × 10^5^ for *T. b. brucei* s427, B48, *T. equiperdum*; 2 × 10^6^ for *L. mexicana,* 4 × 10^5^ for *T. evansi*, 5 × 10^5^ for *T. congolense*), of which 100 μL was added to all the wells in the plate, making a final top concentration of 100 μg/mL drug (200 μg/mL for *L. mexicana*). This was followed by the incubation of the plates at 37 °C/5% CO_2_ (s427, B48, *T. evansi* and *T. equiperdum*), 34 °C/5% CO_2_ (*T. congolense*), or 27 °C (*L. mexicana*) for 48 h (72 h for *L. mexicana*), before the addition of the resazurin dye (20 µL of 125 mg/L), and a further incubation under the same conditions for 24 h (48 h for *L. mexicana*). The extended incubation period for *L. mexicana* was due to the slower metabolism of the dye by *Leishmania* promastigotes [56]. The fluorescence of the wells was read using a FLUOstar OPTIMA Fluorimeter (BMG Labtech, Durham, NC, USA) at wavelengths 544 nm for excitation and 590 nm for emission and a gain of 1250. The fluorescence values were analyzed using GraphPad Prism 9 or higher, GraphPad Software Inc., San Diego, CA, USA, plotting the data (using an equation) to a sigmoidal dose–response curve with variable slope, after which the EC_50_ (half maximal effective concentration of isolated compounds or control drug that induces a response halfway between the baseline and maximum after 72 h exposure time) values were determined. *p*-values were calculated using the unpaired Student’s t-test. All experiments were performed on at least three different fully independent occasions.

### 3.6. Toxicity Assays

The assay assessing the toxicity of the isolated compounds to mammalian cells was carried out on human embryonic kidney (HEK 293) cells, as previously described [54]. Exactly 100 μL (3 × 10^5^ cell/mL) of a cell suspension grown in a vented flask (~80% confluence) under incubation conditions of 37 °C/5% CO_2_ was added to each well of a 96-well plate. The plate was incubated for 24 h cytoadherence, after which 100 µL of a serially diluted drug was added (prepared in a separate sterile plate over one row). Phenylarsine oxide (PAO (Sigma)) was used as the positive control. The cells were then incubated for a further 30 h before the addition of 10 µL sterile Alamar Blue solution (125.0 mg/mL resazurin sodium salt (Sigma) in distilled water), followed by a further 24 h incubation. Fluorescence measurements and data analysis were performed as for the anti-parasite assays. The selectivity index (SI) was calculated as EC_50_ (HEK)/EC_50_ (parasite). All experiments were performed on at least three different fully independent occasions.

## 4. Conclusions

Tarkalynin A and B, as well as taccalonolide A and its propanoate, were isolated from *Tacca leontopetaloides*, while taraxerol acetate, dihydrochelerythrin and fagaramide were isolated from *Zanthoxylum zanthoxyloides*. Dihydrochelerythrin (**6**) showed the highest anti-kinetoplastid activity across the board (all EC_50_ between 1.4 and 8.3 µM), and the highest selectivity index values (SI = 87 for *T. evansi*). Furthermore, fagaramide (**7**) also displayed broad anti-kinetoplastid activity, albeit somewhat less potently (EC_50_ between 10.9 and 34.4 µM). Tarkalynin A (**1**) and taraxerol acetate (**5**) also displayed activity with an EC_50_ of ~20 µM against *T. equiperdum*. No loss of activity was observed towards the multi-drug-resistant *T. b. brucei* clone B48. The study reveals that compounds derived from *Z. zanthoxyloides*, in particular, exhibit genuine anti-kinetoplastid properties, and these should now be further investigated.

## Figures and Tables

**Table 1:** NMR data (400 Hz, in CDCl_3_) (δ, ppm) for compounds **1** and **2**.

Position	Compound 1		Compound 2	
	^1^H (δ ppm, m, *J* (Hz))	^13^C (m)	^1^H (δ ppm, m, *J* (Hz))	^13^C (m)
1	-	197.9 (C)	-	201.4 (C)
2	3.46 (dd, 14.9, 2.9) 2.86 (dd, 14.9, 7.6)	30.5 (CH_2_)	3.33 (m)	38.8 (CH_2_)
3	5.18 (dd, 7.6, 2.9)	75.3 (CH)	2.95 (dd, 6.6, 4.4)	16.6 (CH_2_)
1′	-	125.9 (C)	-	131.4 (C)
2′	8.09 (d, 9.0)	131.5 (CH)	7.63 (dd, 8.3, 1.8)	125.0 (CH)
3′	7.04 (d, 9.0)	114.0 (CH)	6.84 (d, 8.3)	107.9 (CH)
4′	-	164.4 (C)	-	152.3 (C)
5′	7.04 (d, 9.0)	114.0 (CH)	-	147.7 (C)
6′	8.09 (d, 9.0)	131.5 (CH)	7.47 (d, 1.8)	108.1 (CH)
1″	-	105.1 (C)	-	108.7 (C)
2″	-	157.7 (C)	-	156.3 (C)
3″	6.23 (d, 2.3)	94.8 (CH)	6.20 (d, 2.4)	94.7 (CH)
4″	-	160.4 (C)	-	159.9 (C)
5″	6.05 (d, 2.3)	91.3 (CH)	6.07 (d, 2.4)	91.3 (CH)
6″	-	158.3 (C)	-	159.3 (C)
O-CH_2_-O	**-**	**-**	6.02 (s)	101.9 (CH_2_)
4′-OCH_3_	3.95 (s)	55.6 (CH_3_)	-	-
4″-OCH_3_	3.79 (s)	55.3 (CH_3_)	3.77 (s)	55.3 (CH_3_)
6″-OCH_3_	3.61 (s)	55.4 (CH_3_)	3.81 (s)	55.5 (CH_3_)
2″-OH	6.13 (s, br)	-	8.80 (s, br)	-

**Table 2:** Effects of isolated compounds on trypanosomes and leishmania. EC_50_ values are average ± SEM of at least three independent determinations.

Compound	*T. b. brucei* s427		*T. b. brucei B48*			*T. evansi*			*T. equiperdum*			*T. congolense*			*L. mexicana*		HEK 293
	EC_50_ (µM)	SI	EC_50_ (µM)	RF	SI	EC_50_ (µM)	RF	SI	EC_50_ (µM)	RF	SI	EC_50_ (µM)	RF	SI	EC_50_ (µM)	SI	EC_50_ (µM)
**1**	35.5 ± 3.6	>8.5	50.2 ± 15.0	1.4	>6.0	46.0 ± 2.4	1.3	>6.5	21.4 ± 3.9	0.60	>14	246 ± 5 ***	6.9	>1.2	581 ± 3 ***	0.52	>300
**3**	64.7 ± 21.2	>2.2	45.4 ± 4.1	0.7	>3.1	66.3 ± 9.5	1.0	>2.2	55.8 ± 0.0	0.86	>2.6	105 ± 41	1.6	>1.4	>285	ND	>143
**4**	53.3 ± 8.6	>2.6	75.1 ± 12.3	1.4	>1.9	82.7 ± 7.4	1.6	>1.7	77.7 ± 10.5	1.5	>1.8	>140	>2.8	ND	>280	ND	>140
**5**	138 ± 61	>3.1	128 ± 5	0.75	>3.4	62.9 ± 1.5	0.4	>6.8	21.3 ± 0.3	0.13	>20	178 ± 14	1.1	2.4	>427	ND	>427
**6**	1.37 ± 0.43	68.0	2.55 ± 0.29	1.8	37.0	1.09 ± 0.06	0.8	87.0	3.09 ± 0.52	2.2	30.4	8.30 ± 0.57 ***	6.0	11.4	5.44 ± 0.57 **	18	94.4 ± 23.2
**7**	17.8 ± 7.3	20.4	10.9 ± 0.4	0.6	33.8	10.9 ± 0.4	0.6	33.0	27.1 ± 0.1	1.5	13.4	34.4 ± 5.7	1.9	10.6	13.3 ± 0.4	27	364 ± 44
PMD	^a^ 0.0066 ± 0.0001 ^b^ 0.0052 ± 0.0006	-	^a^ 0.312 ± 0.0349 *** ^b^ 0.288 ± 0.05 ***	^a^ 47.3 ^b^ 55.4		^a^ 0.016 ± 0.004 ** ^b^ 0.0025 ± 0.0003 **			^a^ 0.0081 ± 0.001 ^b^ 0.0033 ± 0.001			ND			^a^ 1.10 ± 0.03 *** ^b^ 0.76 ± 0.05 ***		ND
DA	ND		ND			ND			ND			^a^ 0.51 ± 0.01 ^b^ 0.46 ± 0.15			ND		ND
PAO	ND		ND			ND			ND			ND			ND		^a^ 0.17 ± 0.01 ^b^ 0.048 ± 0.011

Control: RF = Resistance factor, being EC_50_ (parasite)/ EC_50_(s427 WT). SI = Selectivity index, being EC_50_ (HEK)/EC_50_(parasite). PMD = pentamidine isethionate, DA = diminazene aceturate, PAO = phenyl arsine oxide. ND = not determined. **, *p* < 0.01; ***, *p* < 0.001. **^a^** = EC_50_ of control for **1**, tarkalynin A; **3**, taccalonolide A; **4**, taccalonolide A 12-propanoate; **^b^** = EC_50_ of control for **5**, taraxerol acetate; **6**, chelerythrine; **7**, fagaramide.

## Data Availability

NMR and MS spectra accompanying this paper are provided in the Supplemental Materials.

## Supplementary Materials

The following supporting information can be downloaded at: https://www.mdpi.com/article/10.3390/molecules30112468/s1, Figure S1: Proton NMR tarkalynin A; Figure S2: ^13^C of the tarkalynin A; Figure S3: 2D NMR of tarkalynin A; Figure S4: Mass spectrum tarkalynin A; Figure S5: Proton NMR of tarkalynin B; Figure S6: ^13^C of tarkalynin B; Figure S7: 2D NMR of tarkalynin B; Figure S8: Mass spectrum of tarkalynin B; Figure S9: Proton NMR of taccalonolide A; Figure S10: ^13^C of taccalonolide A; Figure S11: 2D NMR of taccalonolide A; Figure S12: Mass spectrum of taccalonolide A; Figure S13: Proton NMR of taccalonolide A-12-propanoate; Figure S14: ^13^C of taccalonolide A-12-propanoate; Figure S15: 2D NMR of taccalonolide A-12-propanoate; Figure S16: Mass spectrum of taccalonolide A-12-propanoate; Figure S17: Proton NMR of taraxerol acetate; Figure S18: ^13^C of taraxerol acetate; Figure S19: 2D NMR of taraxerol acetate; Figure S20: Proton NMR of dihydrochelerythrin; Figure S21: ^13^C of dihydrochelerythrin; Figure S22: 2D NMR of dihydrochelerythrin; Figure S23: Proton NMR of fagaramide; Figure S24: ^13^C of fagaramide; Figure S25: 2D NMR of fagaramide.
