# Supplementary material for: Antitrypanosomal and Antileishmanial Activities of Tacca leontopetaloides Tubers and Zanthoxylum zanthoxyloides Stem Bark
Source: Molecules. 2025 Jun 5;30(11):2468. doi: 10.3390/molecules30112468 (PMC12156181; doi:10.3390/molecules30112468)
Supplement: Supplementary file 1 [file molecules-30-02468-s001.zip › molecules-3629383-supplementary.pdf]

Supporting information for

Antitrypanosomal and Antileishmanial Activities of *Tacca leontopetaloides* Tubers and *Zanthoxylum zanthoxyloides* Stem Bark

Elizabeth O. Agbo [1,2], John V. Anyam [1,3], Cyprian T. Agber [1,2], Christie A. Adah [1,2], Christopher Agbo [4], Augustina O. Ijeomah [5], Terrumun A. Tor-Anyiin [5], Hamed E. Alkhalaf [6], Aditya Sarode [7], Jamal I. Asseri [6], Alexander I. Gray [8], John O. Igoli [1,5,*] and Harry P. De Koning [6,*]

1 Phytochemistry Research Group, Department of Chemistry, Joseph Sarwuan Tarka University, Makurdi PMB 2373, Benue State, Nigeria; lizzyagbo20885@gmail.com (E.O.A.); johnversh@gmail.com (J.V.A.); agbercyprian@gmail.com (C.T.A.); christieadah5@gmail.com (C.A.A.)
2 Department of Chemistry, Benue State University, Makurdi PMB 102119, Benue State, Nigeria
3 Centre for African Medicinal Plant Research, North-Eastern University, Gombe 771104, Gombe State, Nigeria
4 Department of Microbiology, Joseph Sarwuan Tarka University, Makurdi PMB 2373, Benue State, Nigeria; agbochristopher03@gmail.com
5 Department of Chemistry, Joseph Sarwuan Tarka University, Makurdi PMB 2373, Benue State, Nigeria; tinaijeomah@gmail.com (A.O.I.); toranyiint@yahoo.com (T.A.T.-A.)
6 School of Infection and Immunity, College of Medical, Veterinary and Life Sciences, University of Glasgow, Glasgow G12 8TA, UK; 2932662a@student.gla.ac.uk (H.E.A.); 2264761a@student.gla.ac.uk (J.I.A.)
7 Department of Pure and Applied Chemistry, University of Strathclyde, Glasgow G4 0RE, UK; aditya.sarode@strath.ac.uk
8 Strathclyde Institute of Pharmacy and Biomedical Science, University of Strathclyde, Glasgow G4 0RE, UK; a.i.gray@strath.ac.uk
* Correspondence: igolij@gmail.com (J.O.I.); harry.de-koning@glasgow.ac.uk (H.P.D.K.)

Supplementary Materials

1. Proton NMR tarkalynin A
2. ^{13}C of the tarkalynin A
3. 2D NMR of tarkalynin A
4. Mass spectra tarkalynin A
5. Proton NMR of tarkalynin B
6. ^{13}C of tarkalynin B
7. 2D NMR of tarkalynin B
8. Mass spectrum of tarkalynin B
9. Proton NMR of taccalonolide A
10. ^{13}C of taccalonolide A
11. 2D NMR of taccalonolide A

12. Mass spectrum of taccalonolide A
13. Proton NMR of taccalonolide A 12 propanoate
14. ^{13}C of taccalonolide A 12 propanoate
15. 2D NMR of taccalonolide A 12 propanoate
16. Mass spectrum of taccalonolide A 12 propanoate
17. Proton NMR of taraxerol acetate
18. ^{13}C of taraxerol acetate
19. 2D NMR of taraxerol acetate
20. Proton NMR of dihydrochelerythrin
21. ^{13}C of dihydrochelerythrin
22. 2D NMR of dihydrochelerythrin
23. Proton NMR of fagaramide
24. ^{13}C of fagaramide
25. 2D NMR of fagaramide

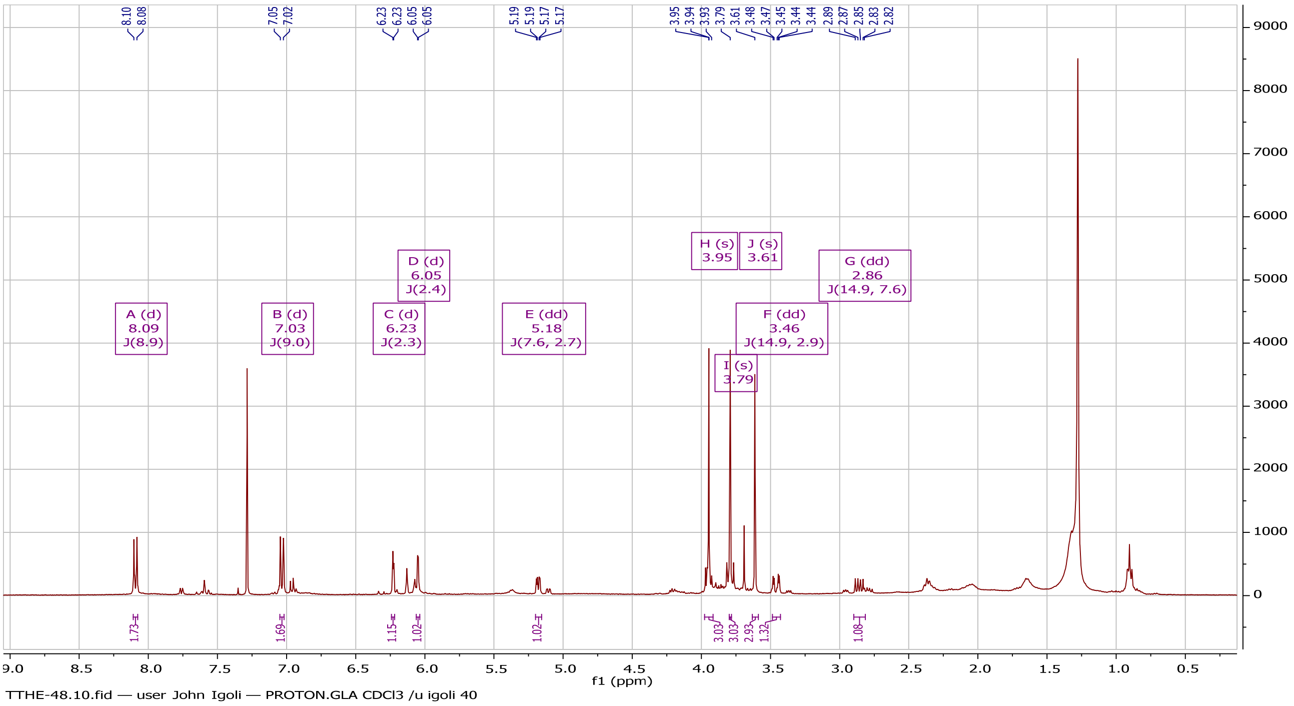

Figure S1: ^{1}H-NMR of tarkalynin A **(1)**

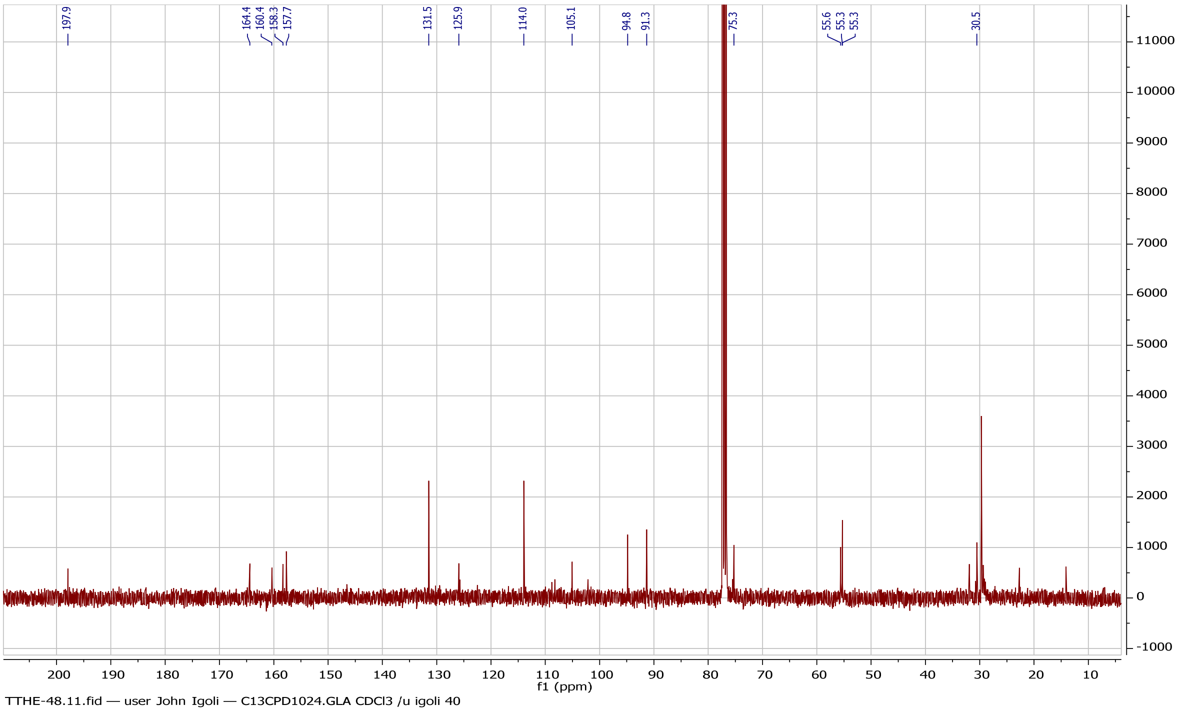

Figure S2: ^{13}C-NMR of tarkalynin A **(1)**

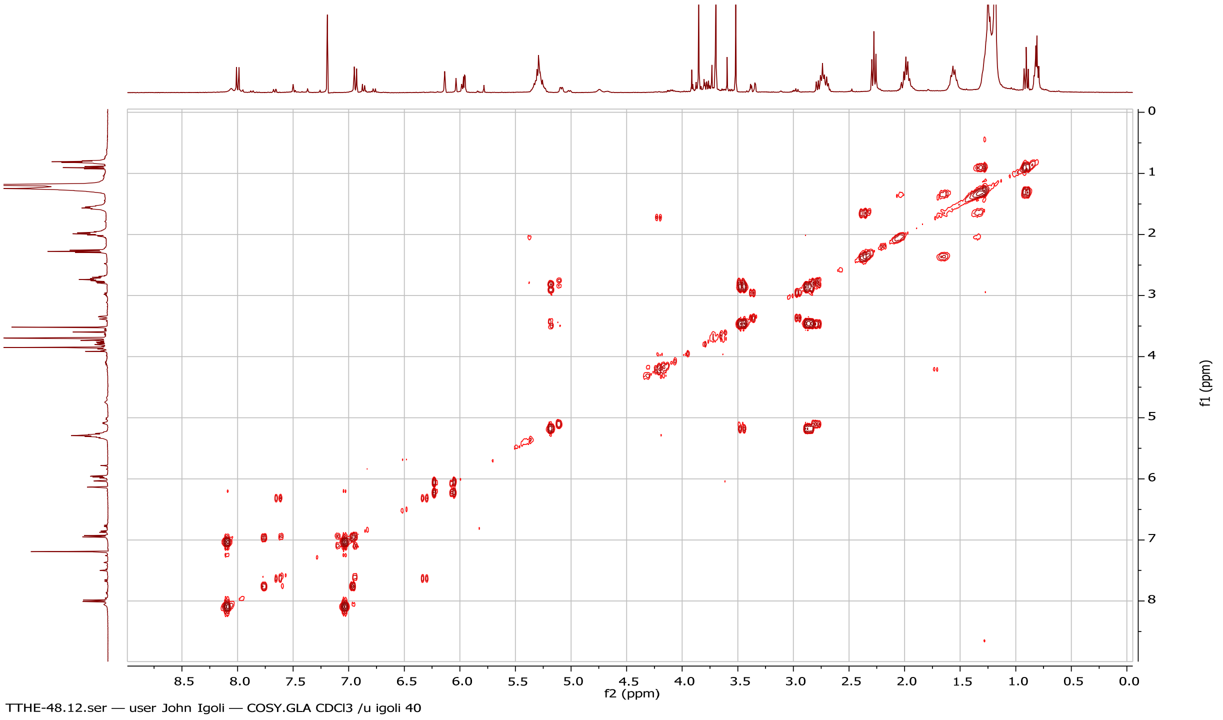

Figure S3A: COSY of tarkalynin A **(1)**

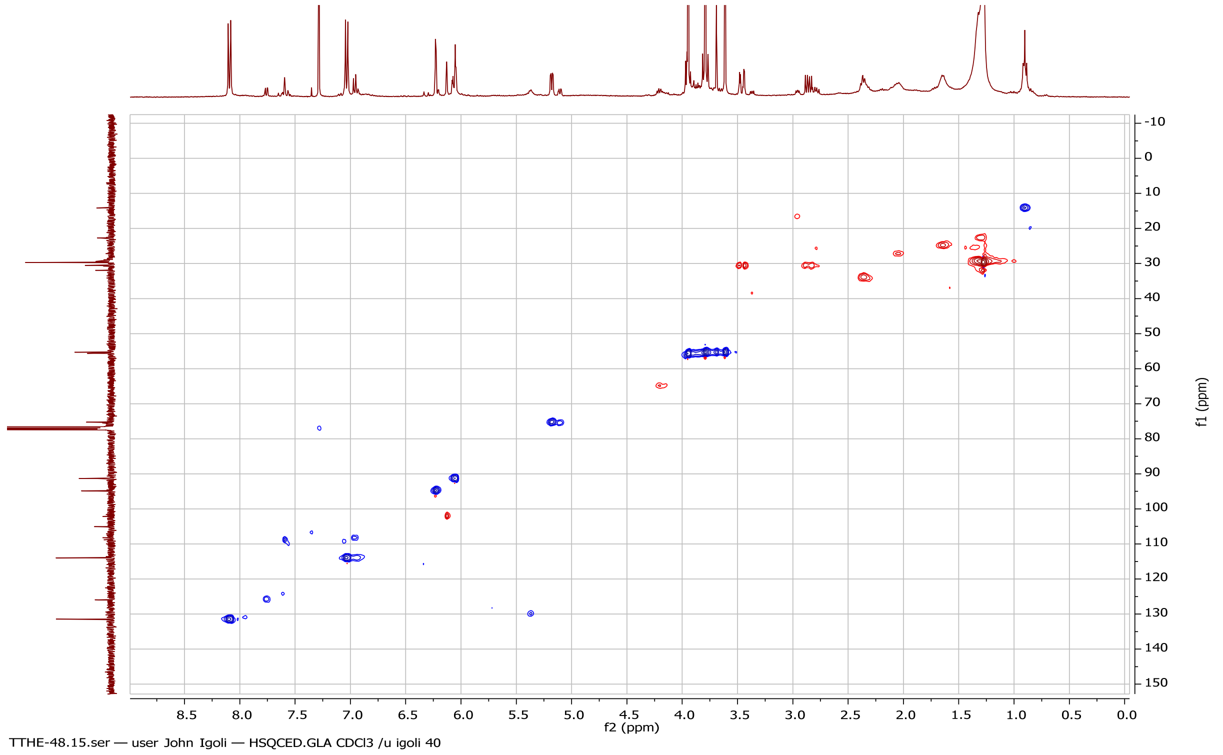

Figure S3B: HSQC of tarkalynin A **(1)**

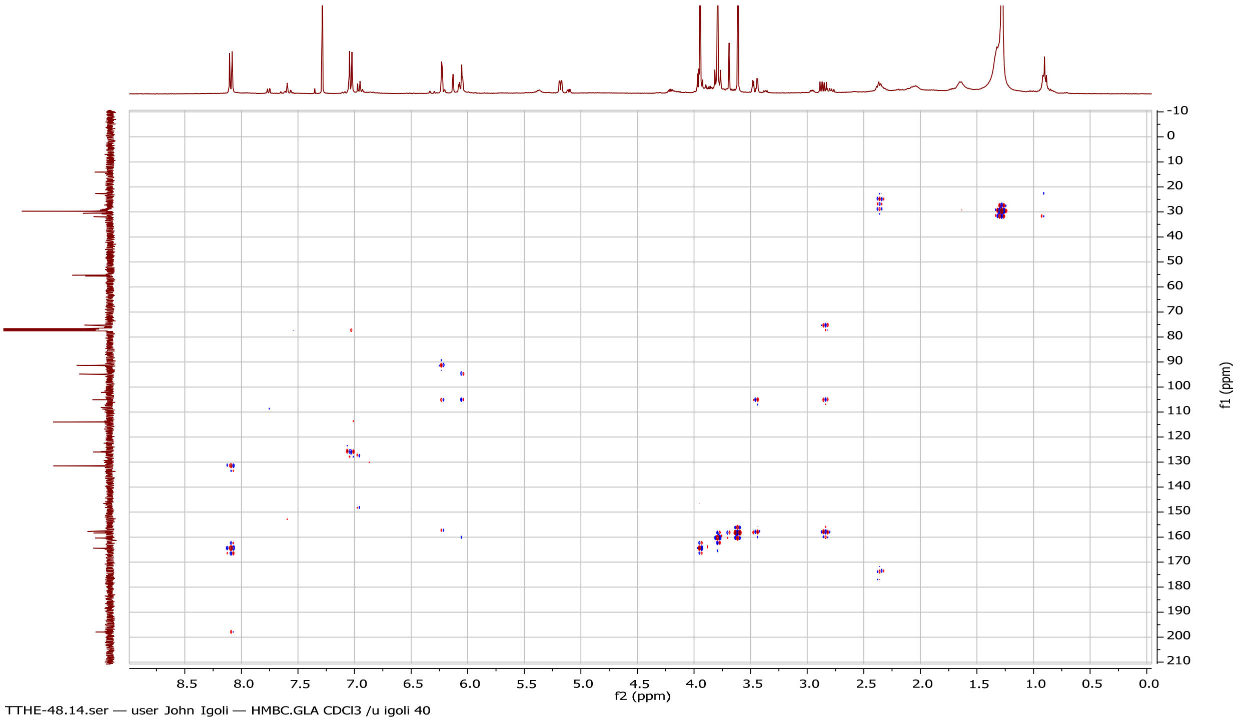

Figure S3C: HMBC of tarkalynin A **(1)**

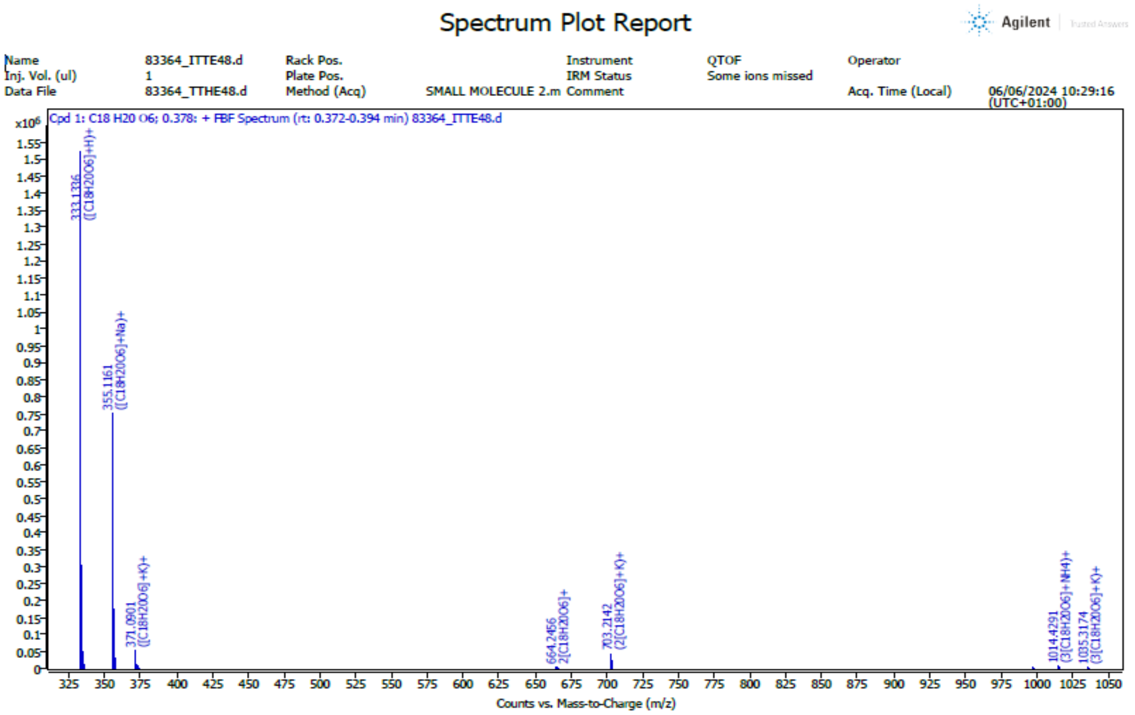

Figure S4: MS of tarkalynin A **(1).** Found $[M+H]^+$ = 333.1336 (Calc. 333.1338) corresponding to $C_{18}H_{20}O_6$

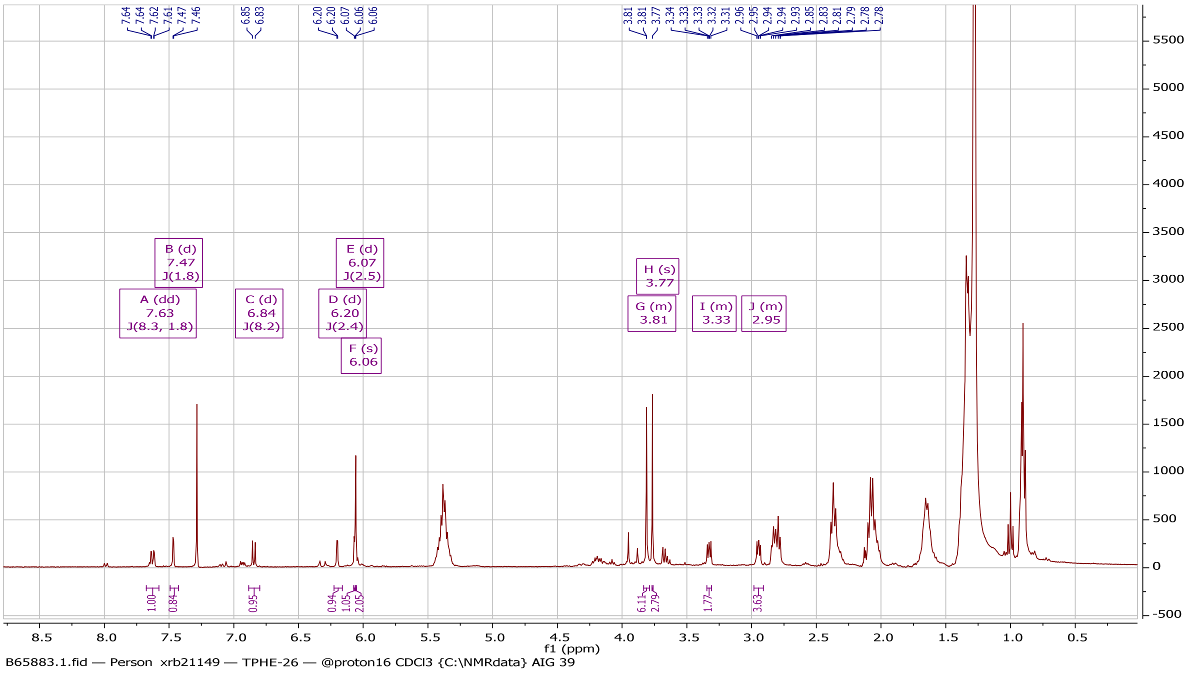

Figure S5: ^{1}H-NMR of tarkalynin B **(2)**

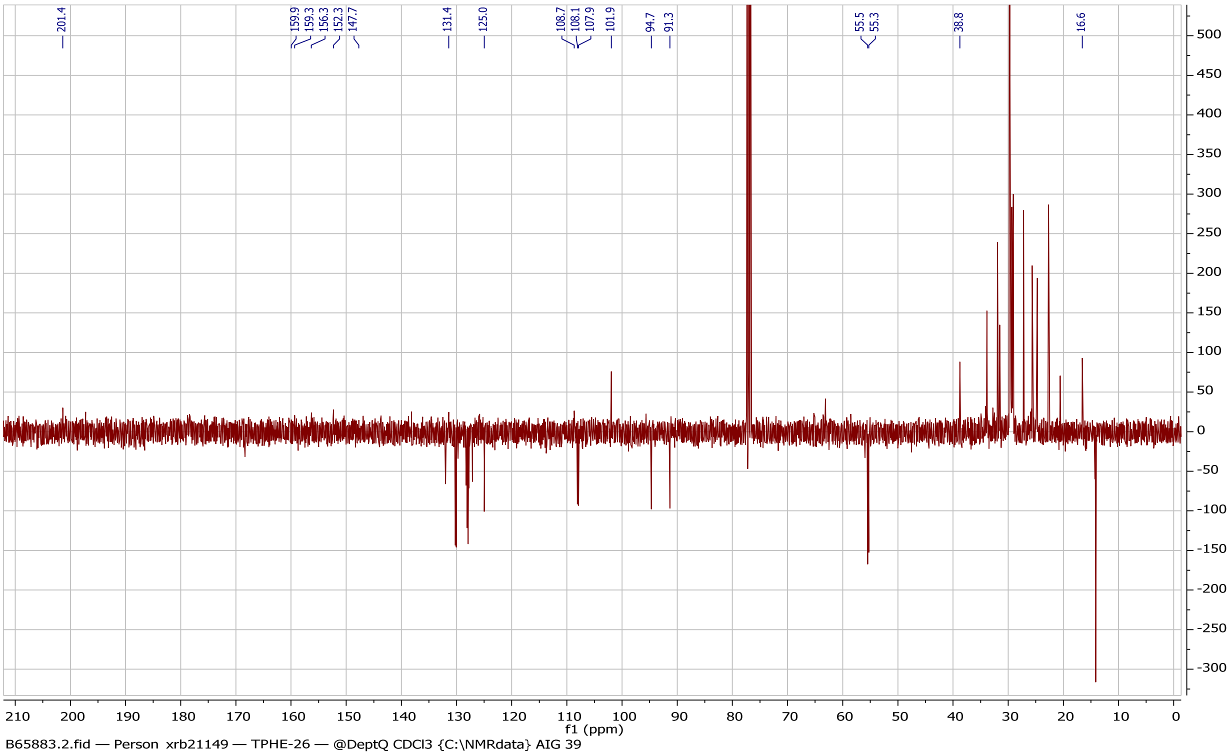

Figure S6: ^{13}C-NMR of tarkalynin B **(2)**

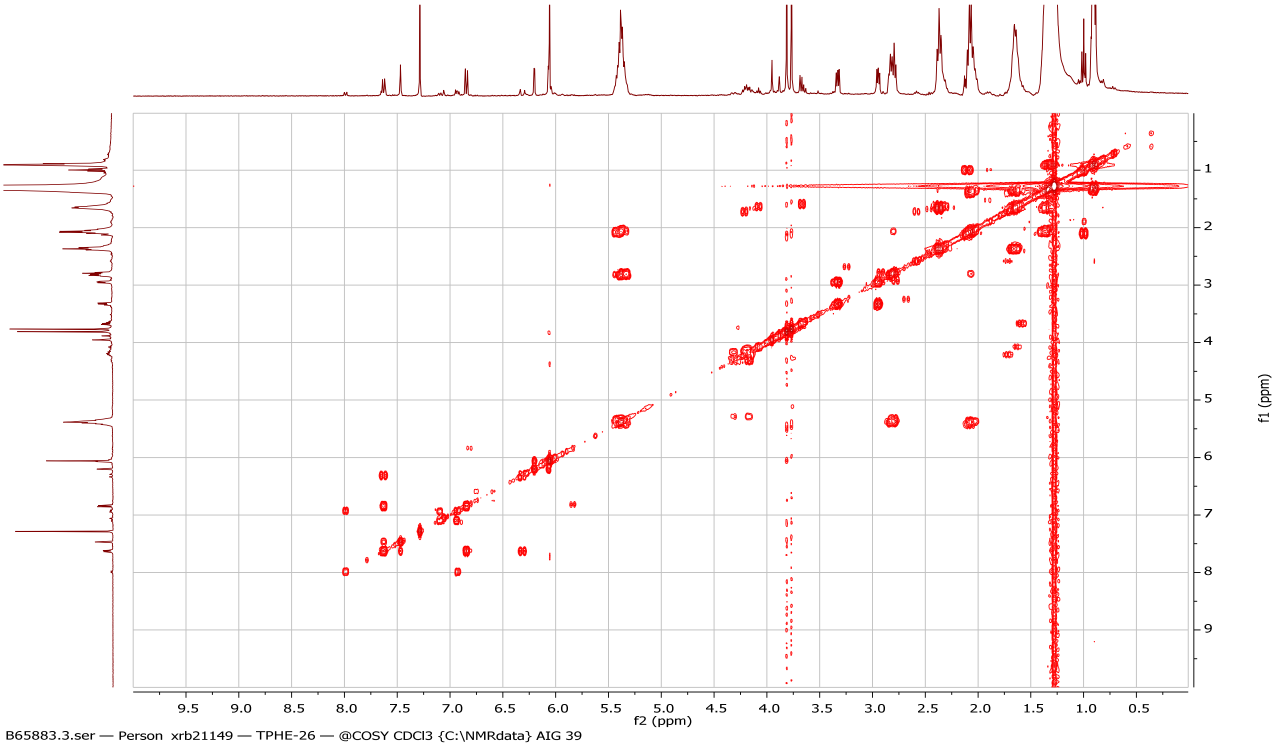

Figure S7A: COSY of tarkalynin B **(2)**

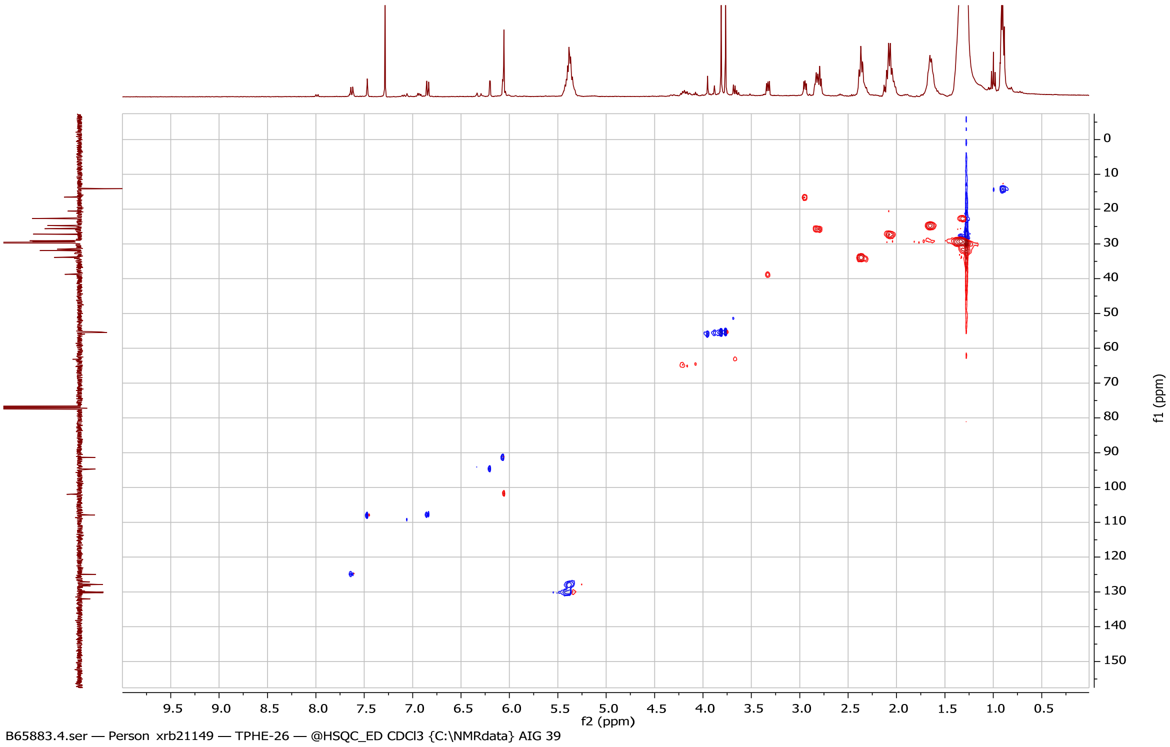

Figure S7B: HSQC of tarkalynin B **(2)**

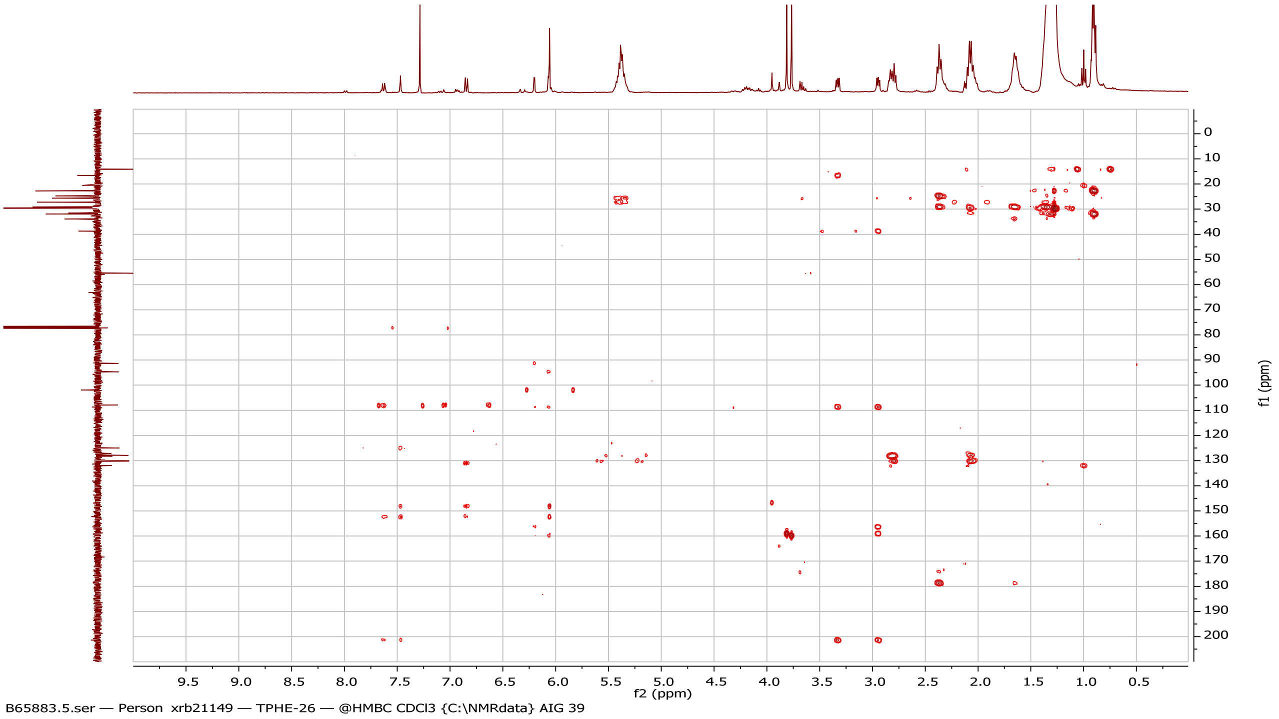

Figure S7C: HMBC of tarkalynin B **(2)**

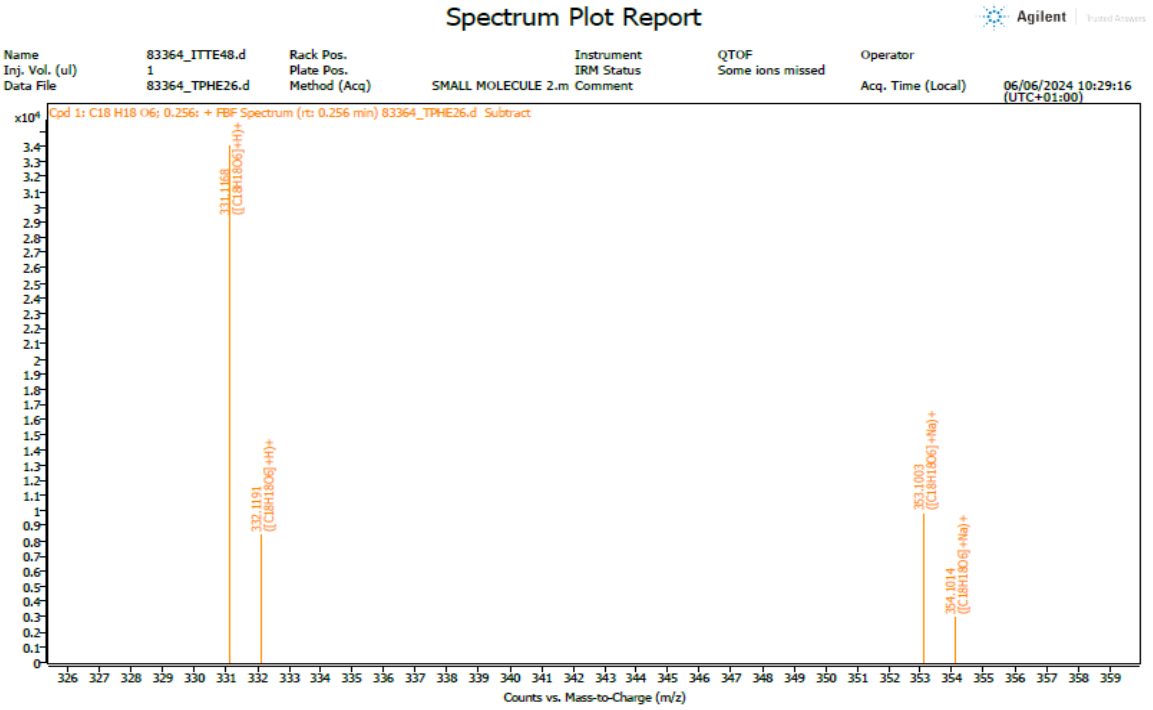

Figure S8: MS of tarkalynin B **(2).** Found $[M+H]^+$ = 331.1168 (Calc. 331.1182), corresponding to $C_{18}H_{18}O_6$

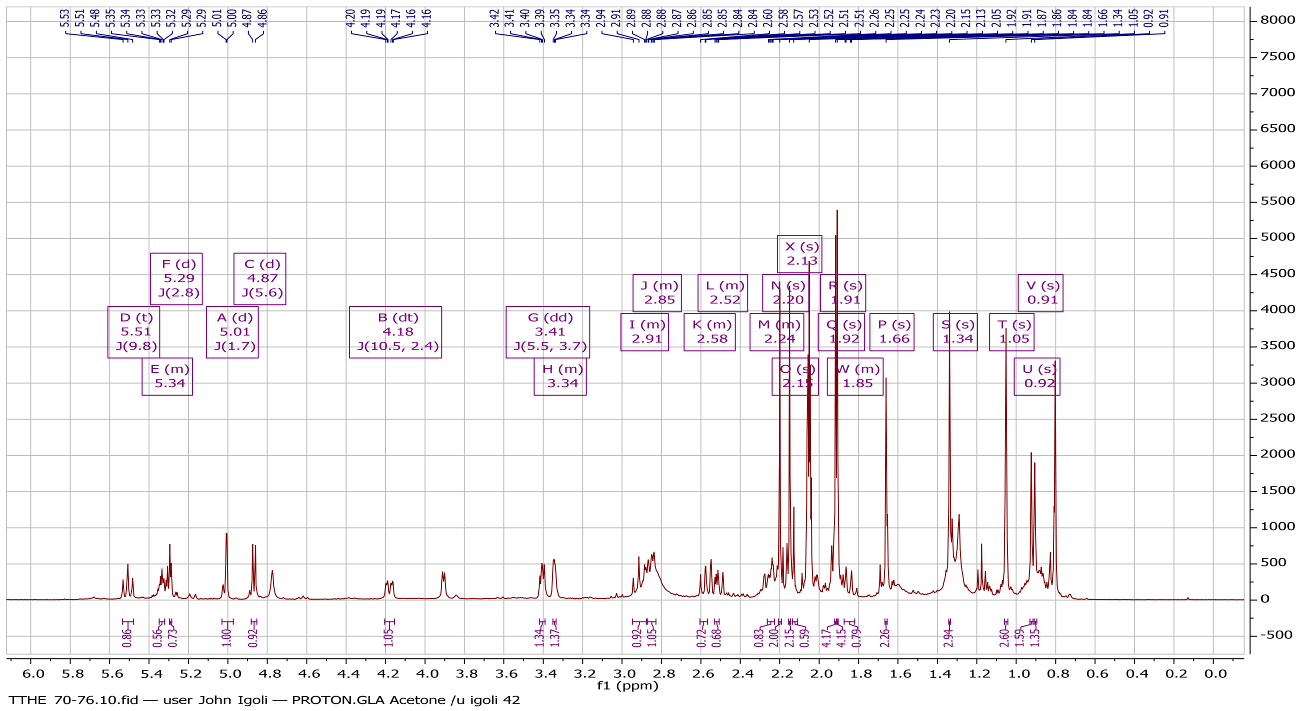

Figure S9: ^{1}H-NMR of taccalonolide A **(3)**

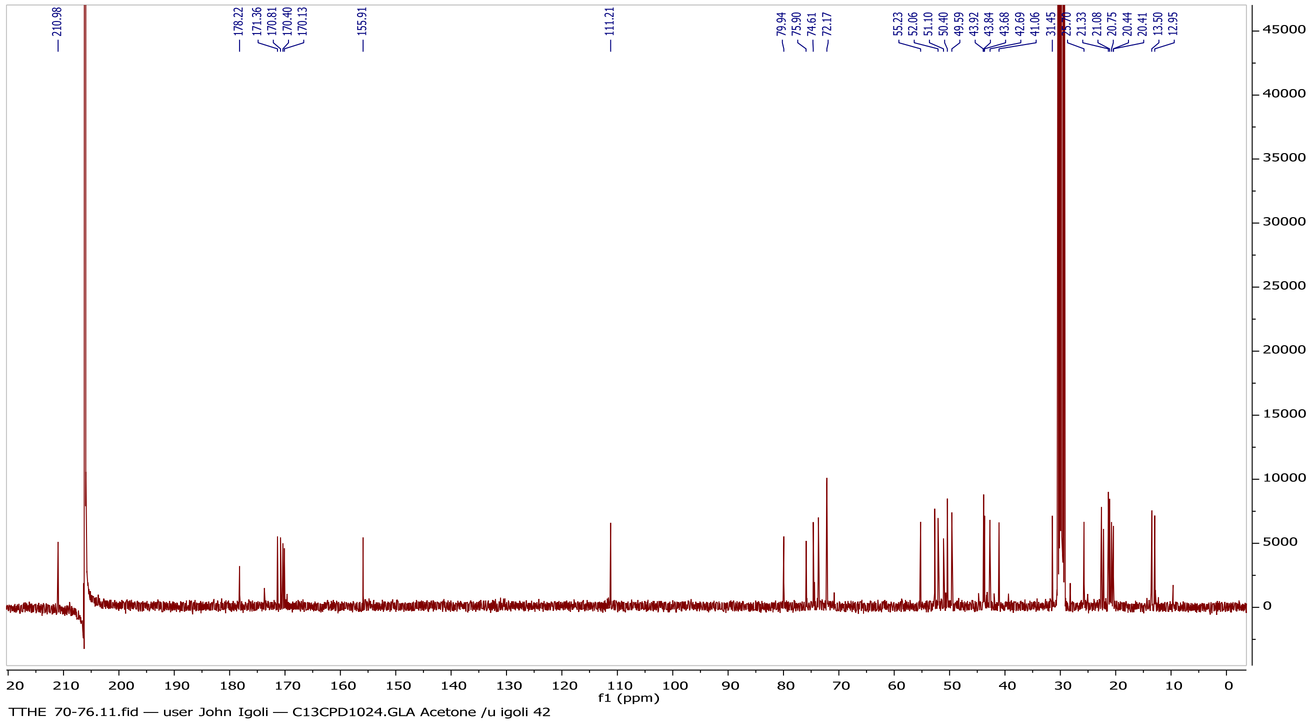

Figure S10: ^{13}C-NMR of taccalonolide A **(3)**

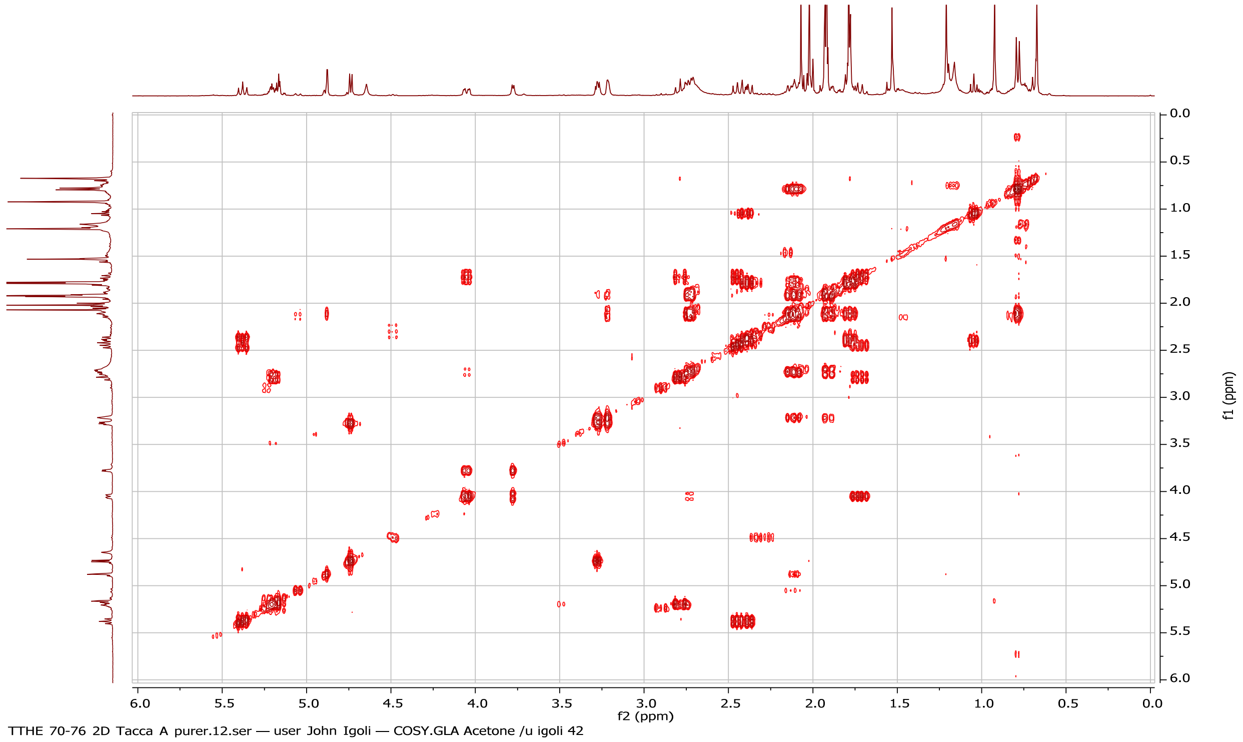

Figure S11A: COSY of taccalonolide A **(3)**

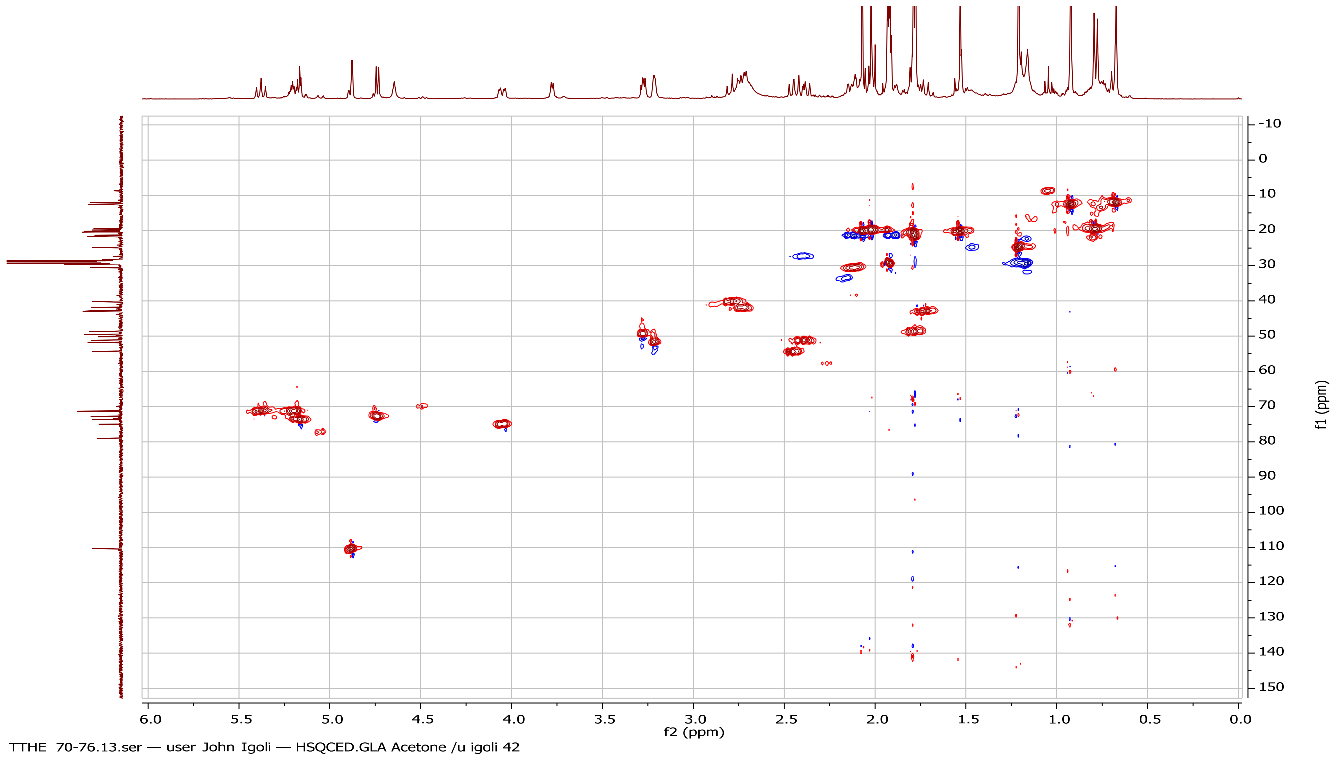

Figure S11B: HSQC of taccalonolide A **(3)**

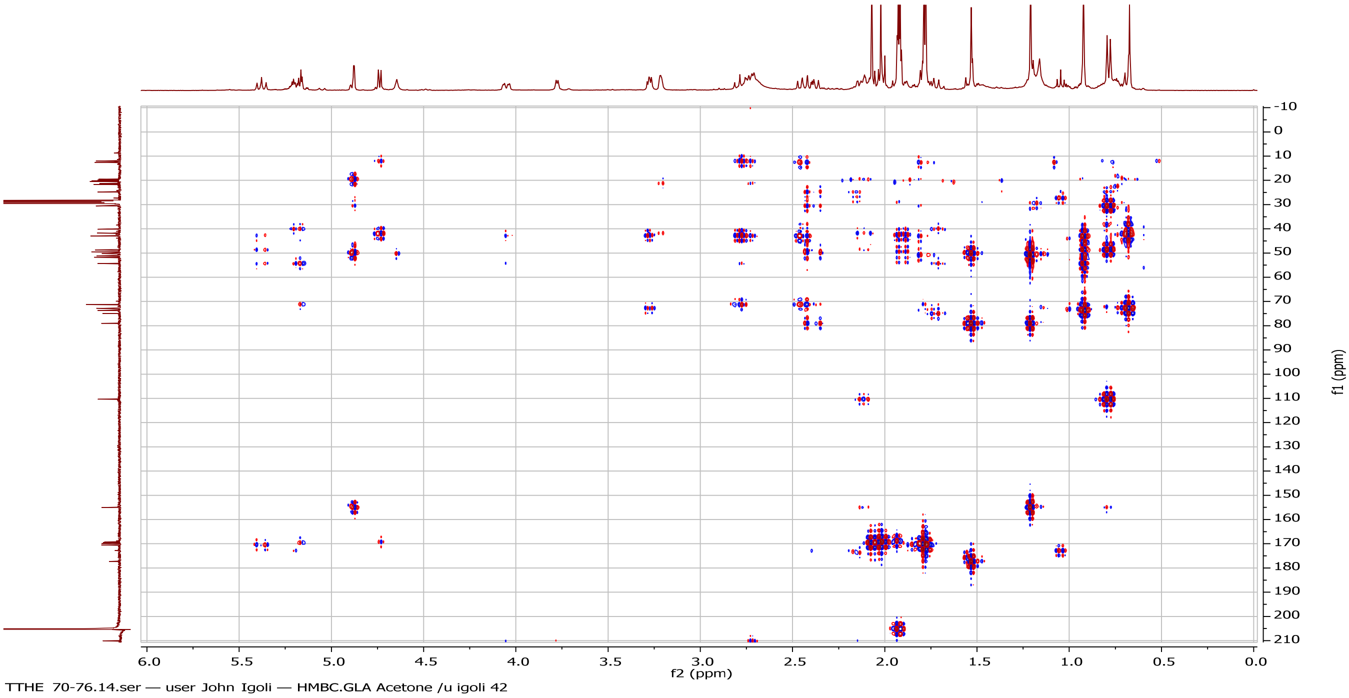

Figure S11C: HMBC of taccalonolide A **(3)**

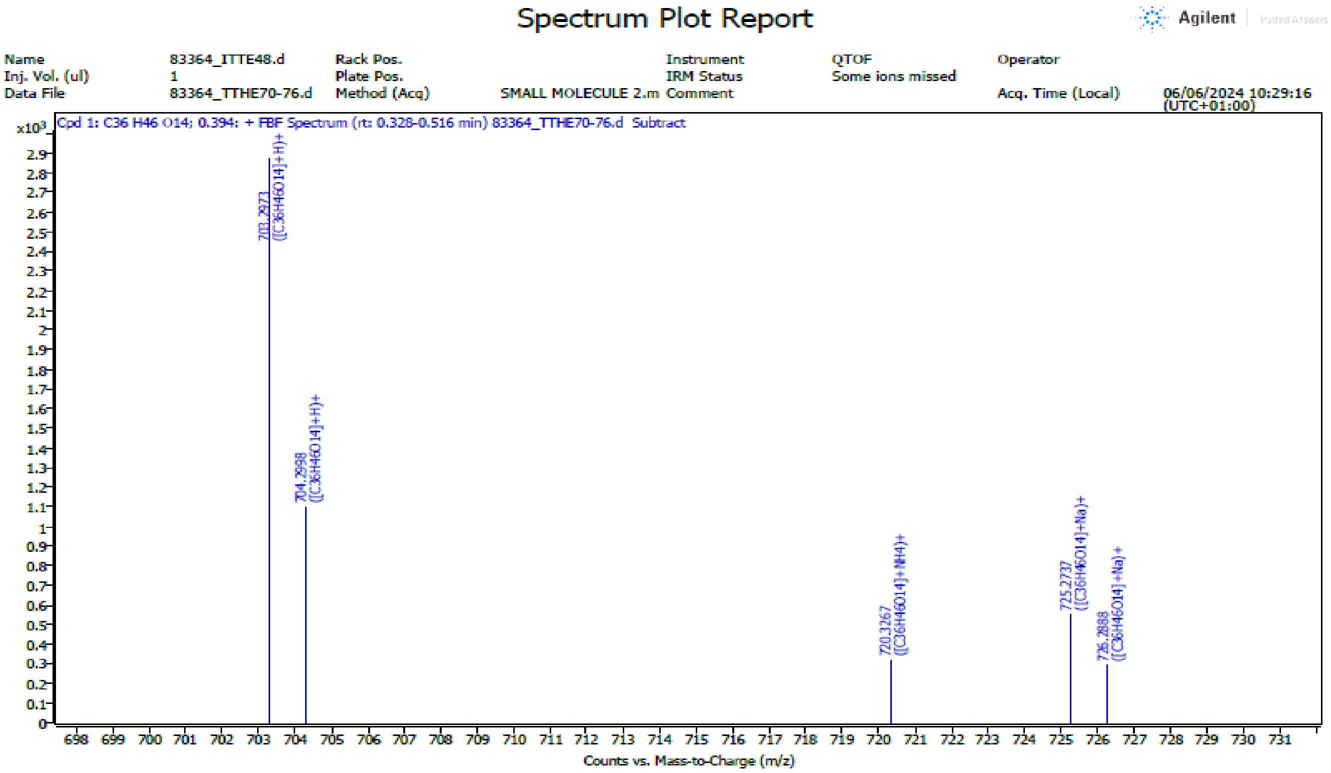

Figure S12: MS of taccalonolide A **(3)**. Found $[M+H]^+$ = 703.2973 (Calc. 703.2966) corresponding to $C_{36}H_{46}O_{14}$

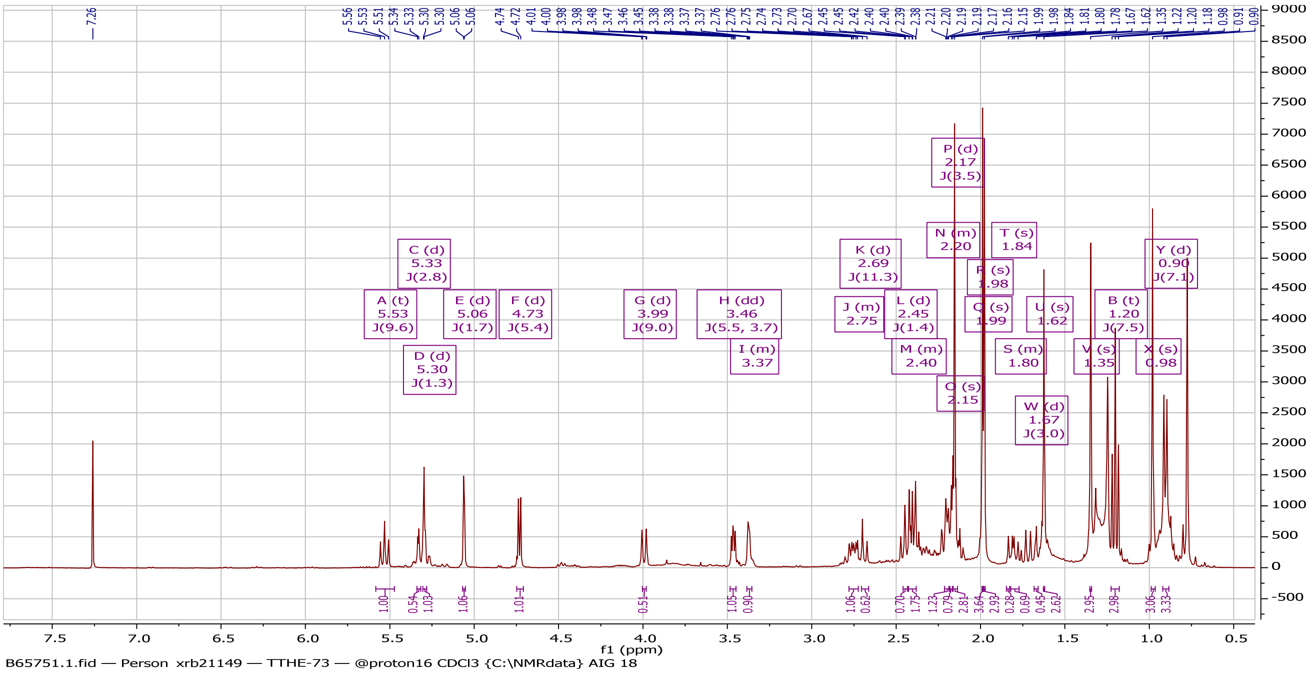

Figure S13: ^{1}H-NMR of taccalonolide A 12 propanoate **(4)**

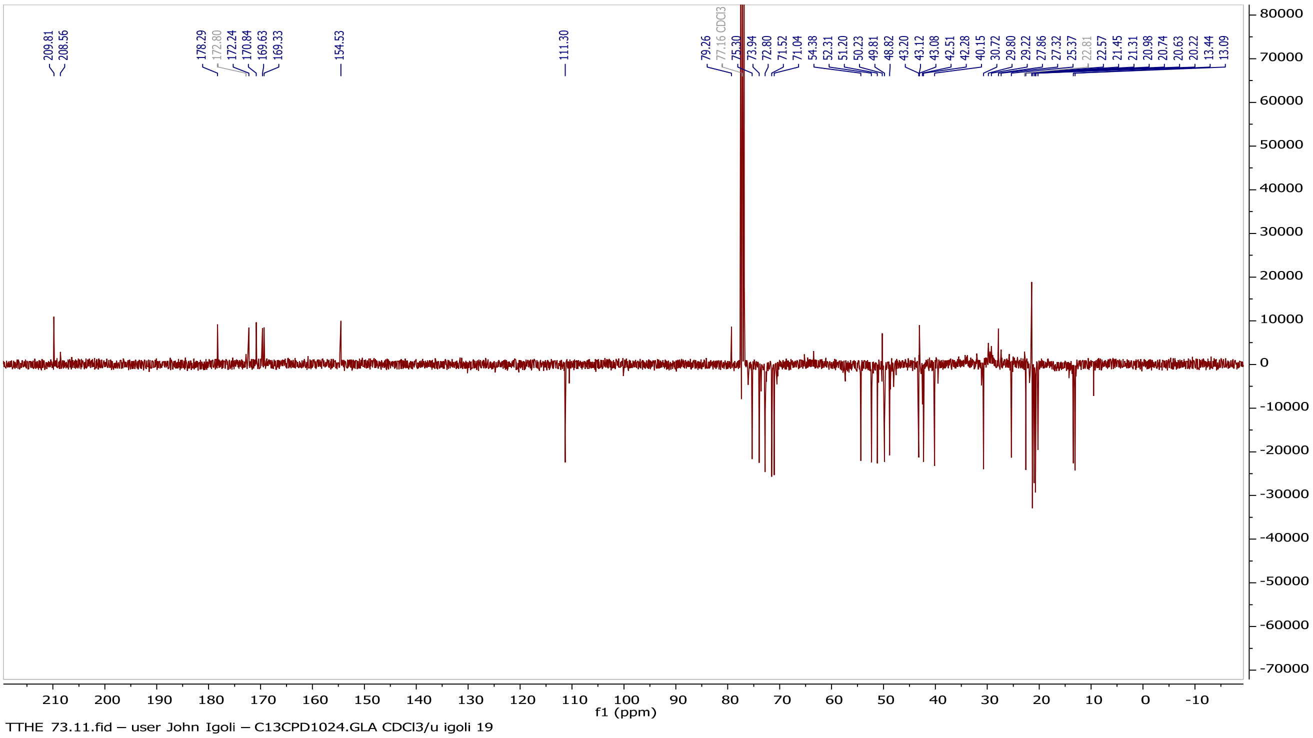

Figure S14: ^{13}C-NMR of taccalonolide A 12 propanoate **(4)**

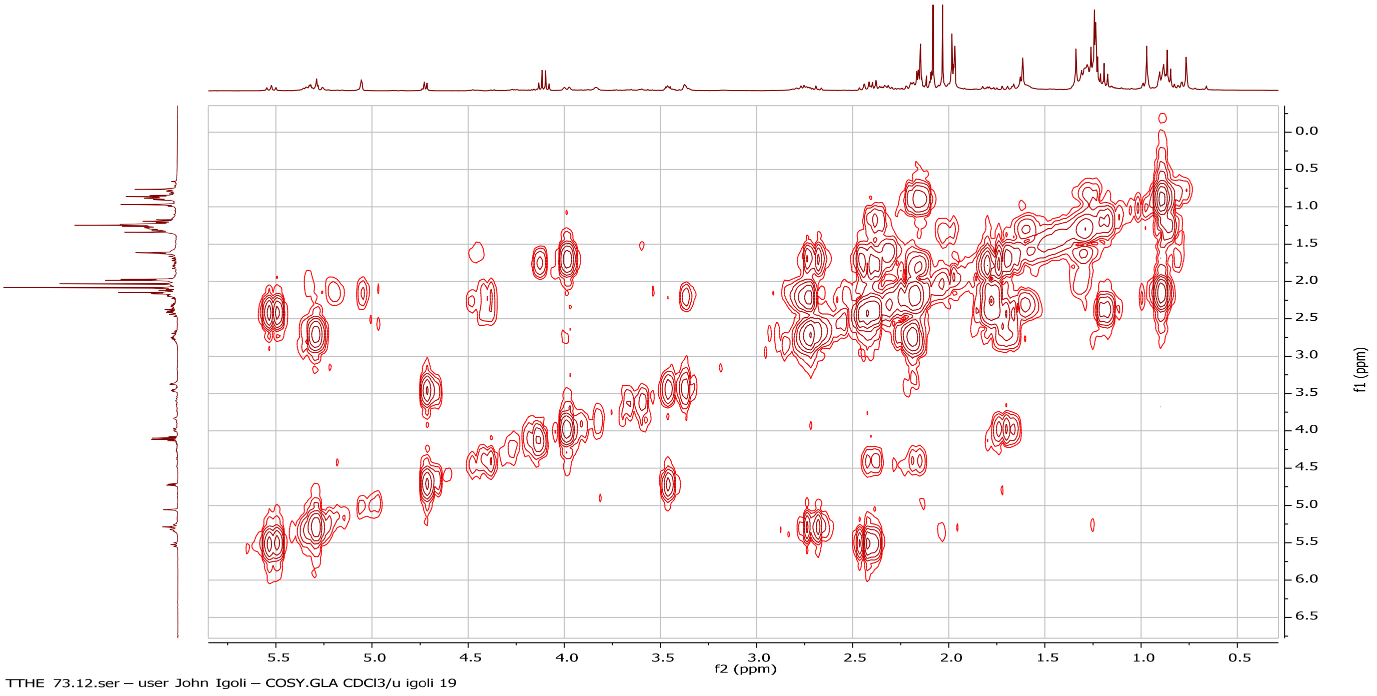

Figure S15A: COSY of taccalonolide A 12 propanoate **(4)**

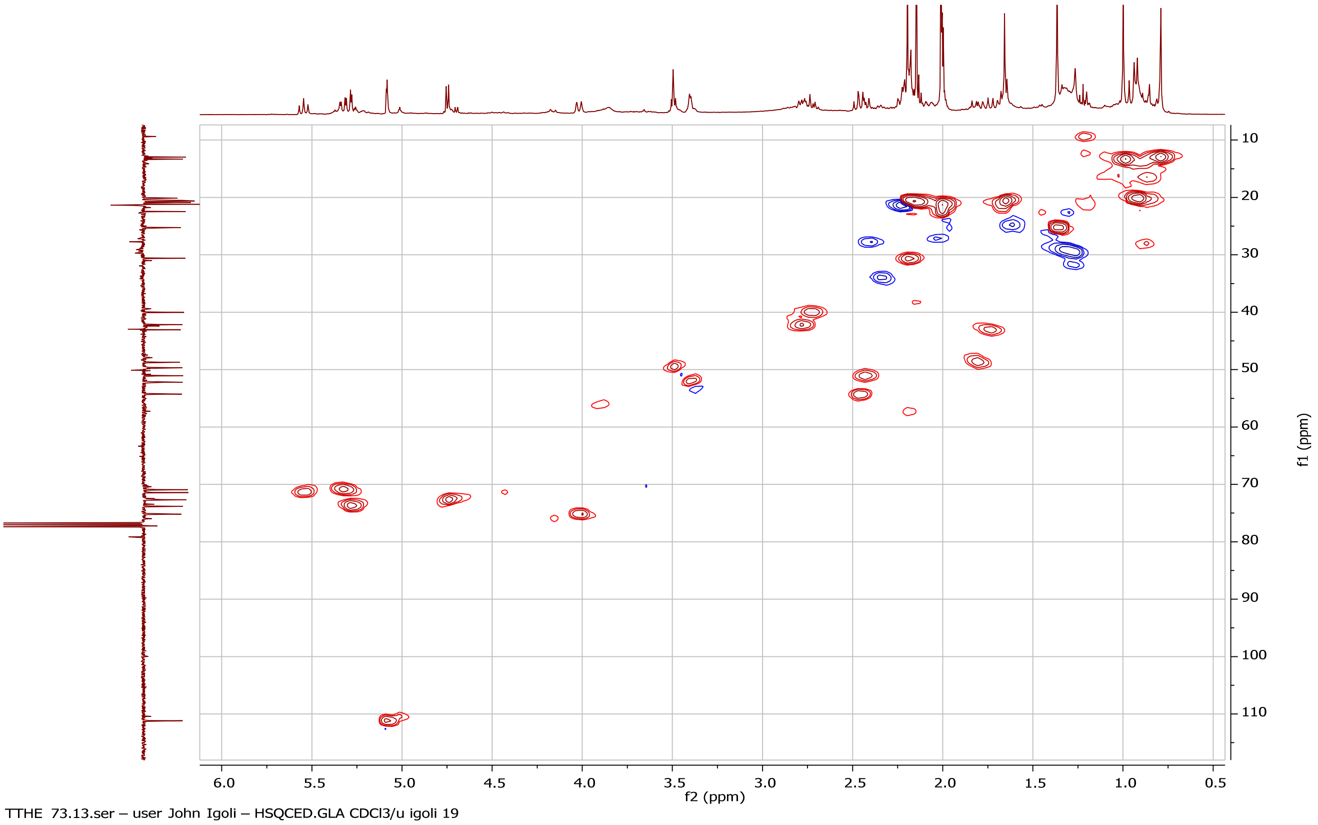

Figure S15B: HSQC of taccalonolide A 12 propanoate **(4)**

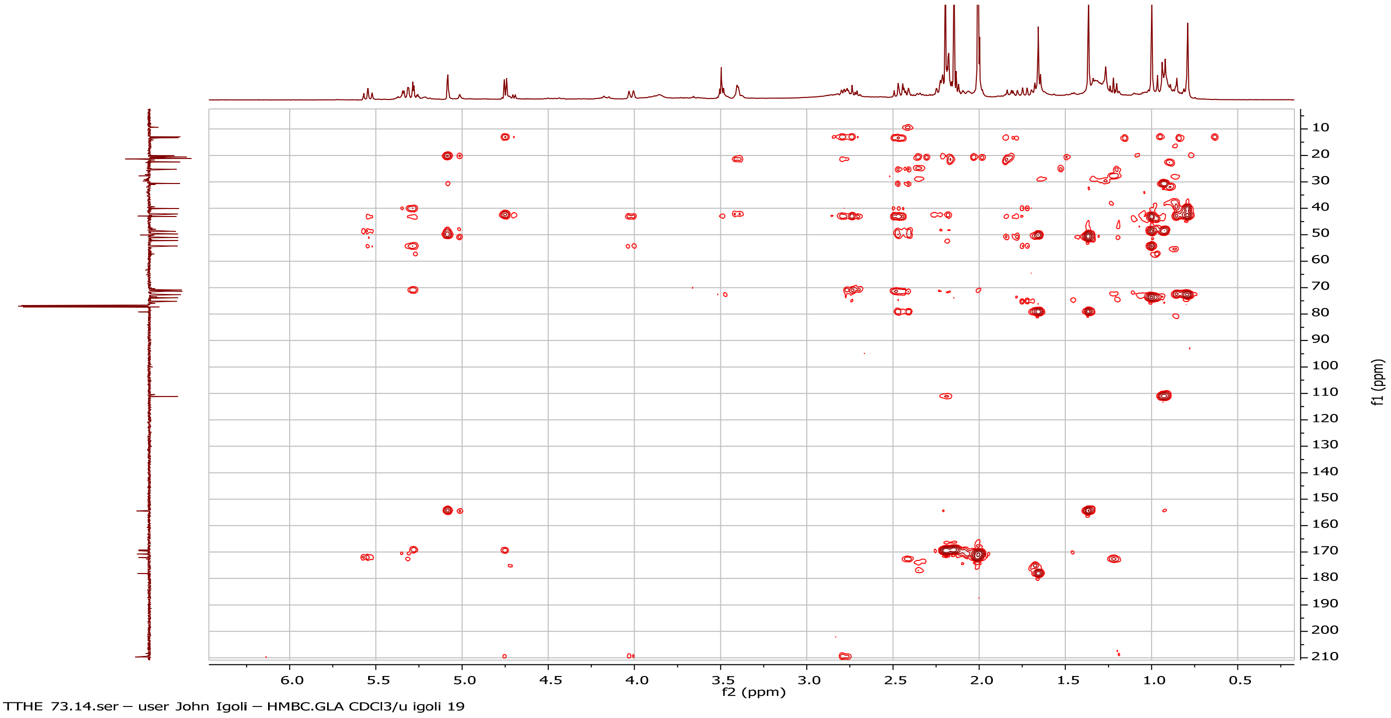

Figure S15C: HMBC of taccalonolide A 12 propanoate **(4)**

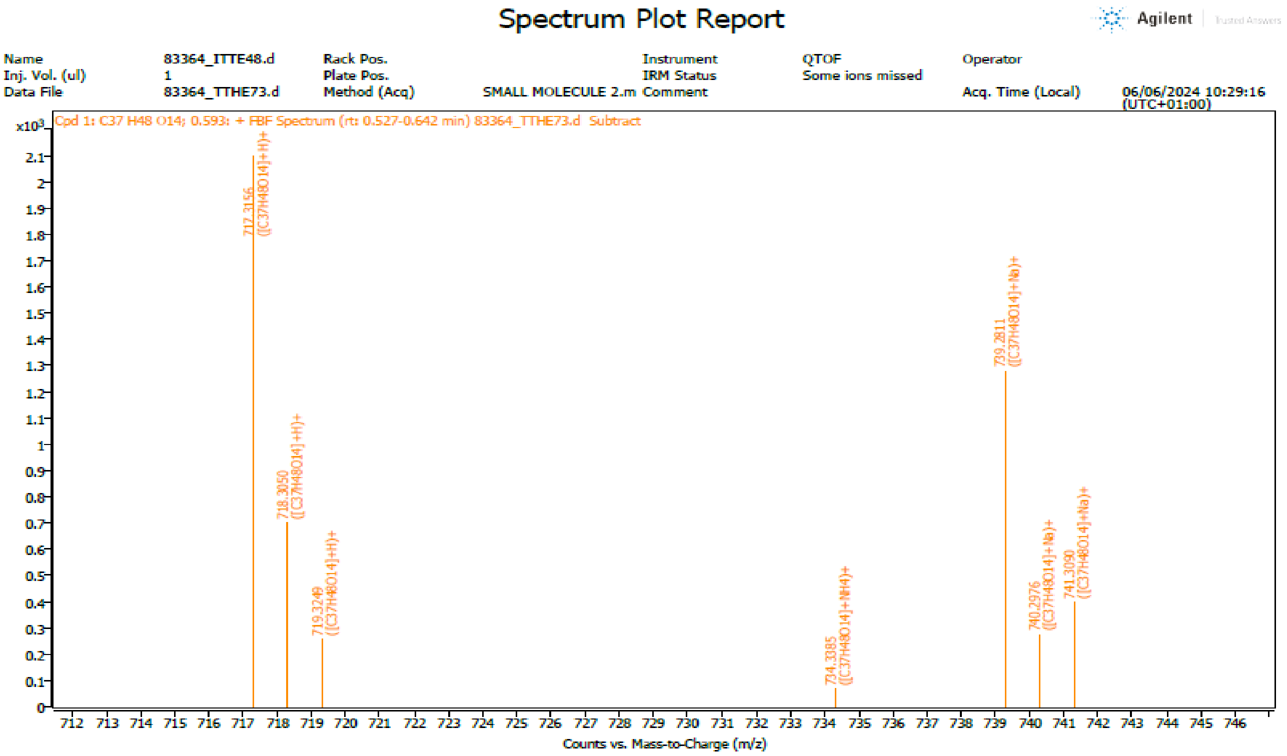

Figure S16: MS of taccalonolide A 12 propanoate **(4)**. Found $[M+H]^+ = 717.3156$ (Calc. 717.3122) corresponding to $C_{37}H_{48}O_{14}$

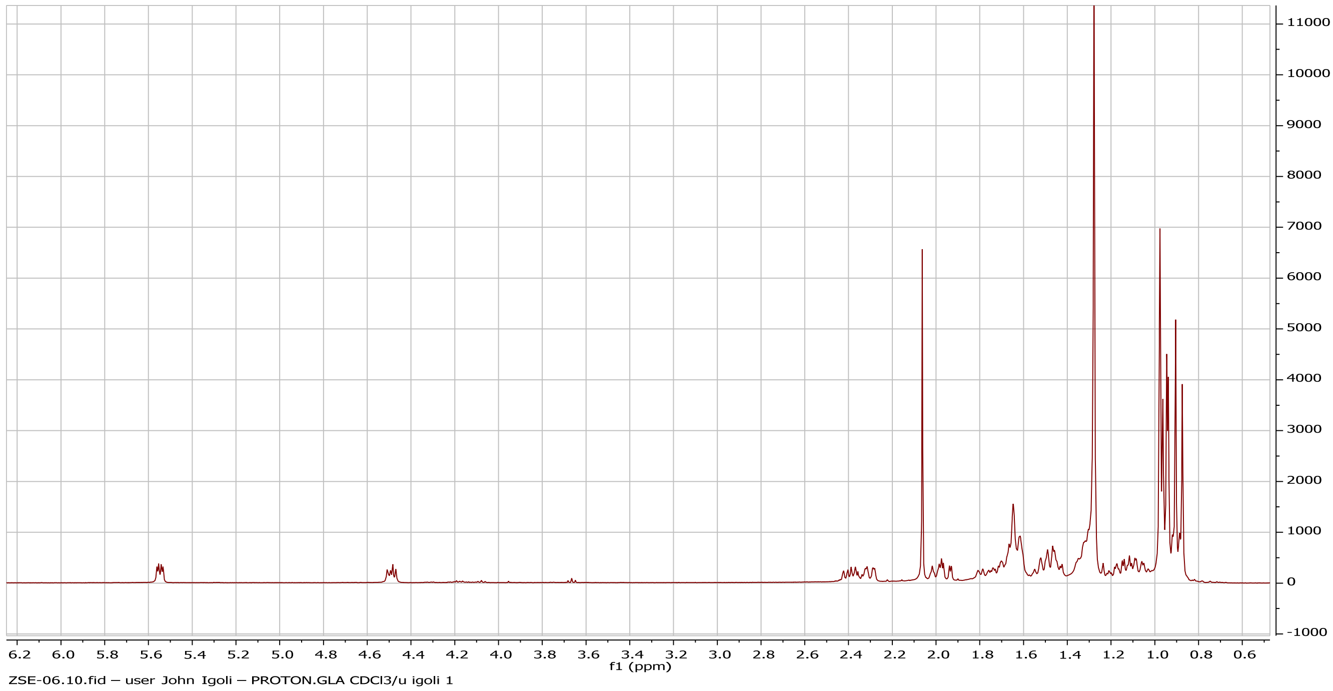

Figure S17: ^{1}H-NMR of taraxerol acetate **(5)**

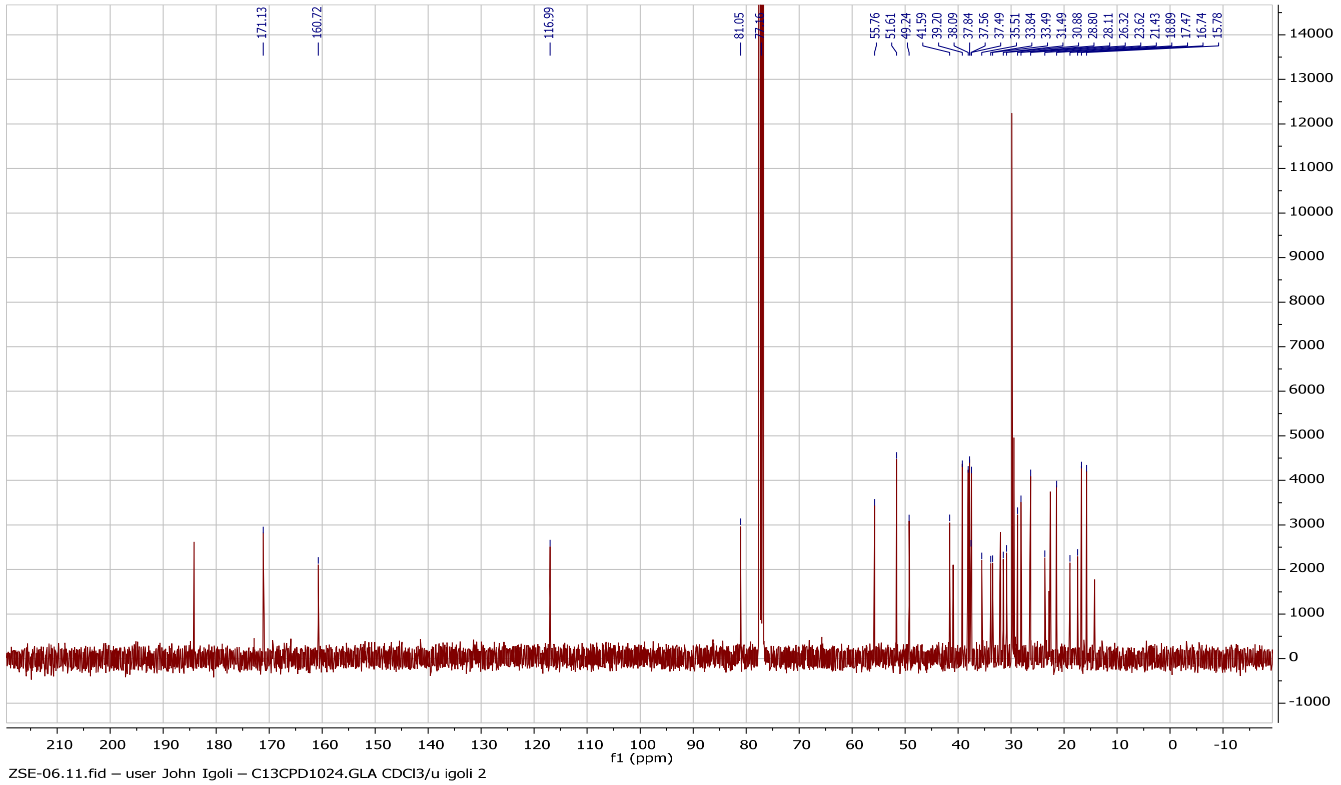

Figure S18: ^{13}C-NMR of taraxerol acetate **(5)**

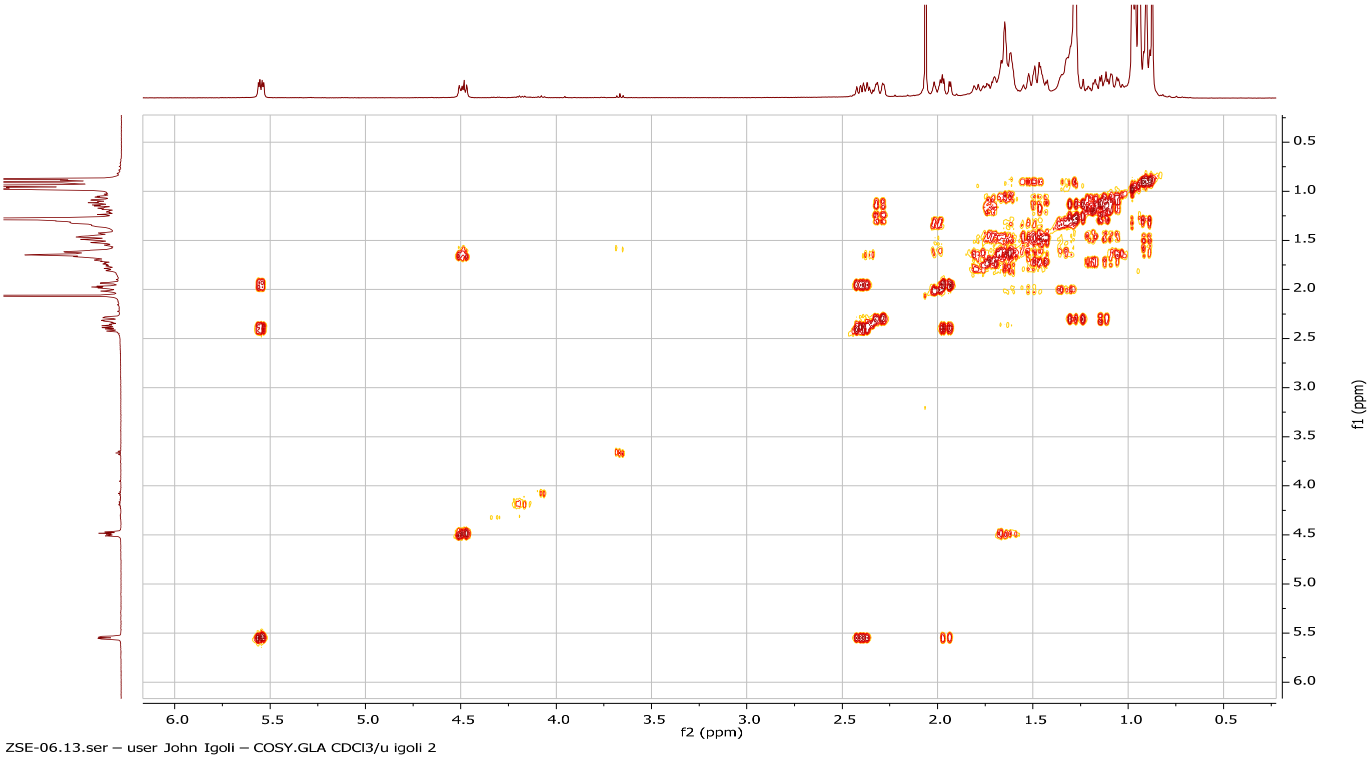

Figure S19A: COSY of taraxerol acetate **(5)**

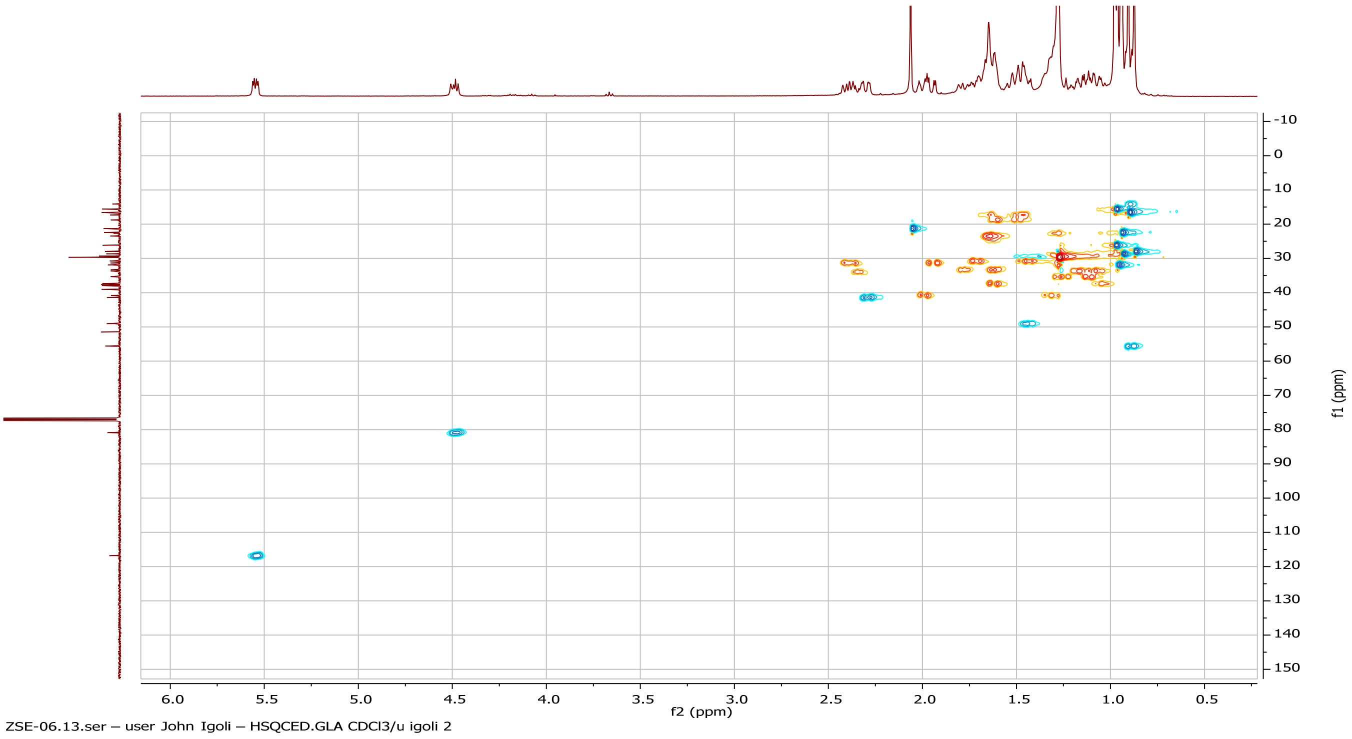

Figure S19B: HSQC of taraxerol acetate **(5)**

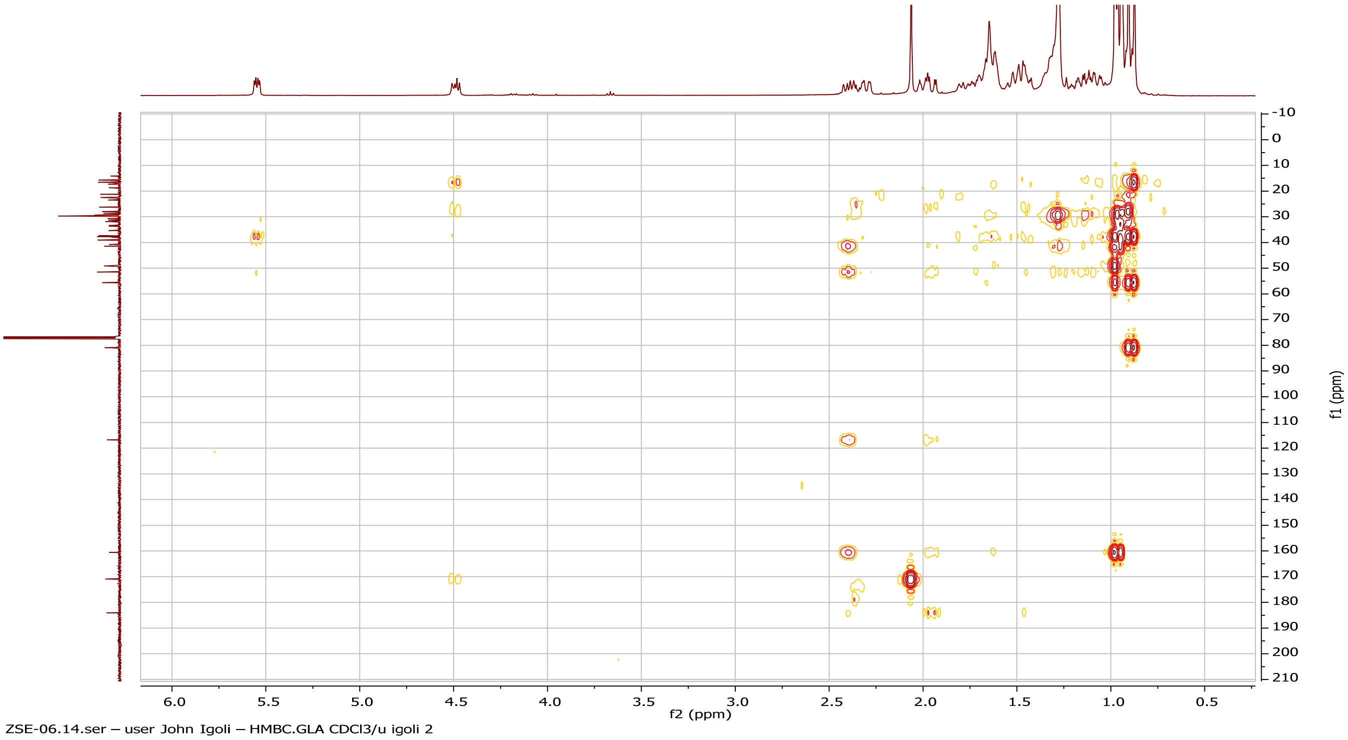

Figure S19C: HMBC of taraxerol acetate **(5)**

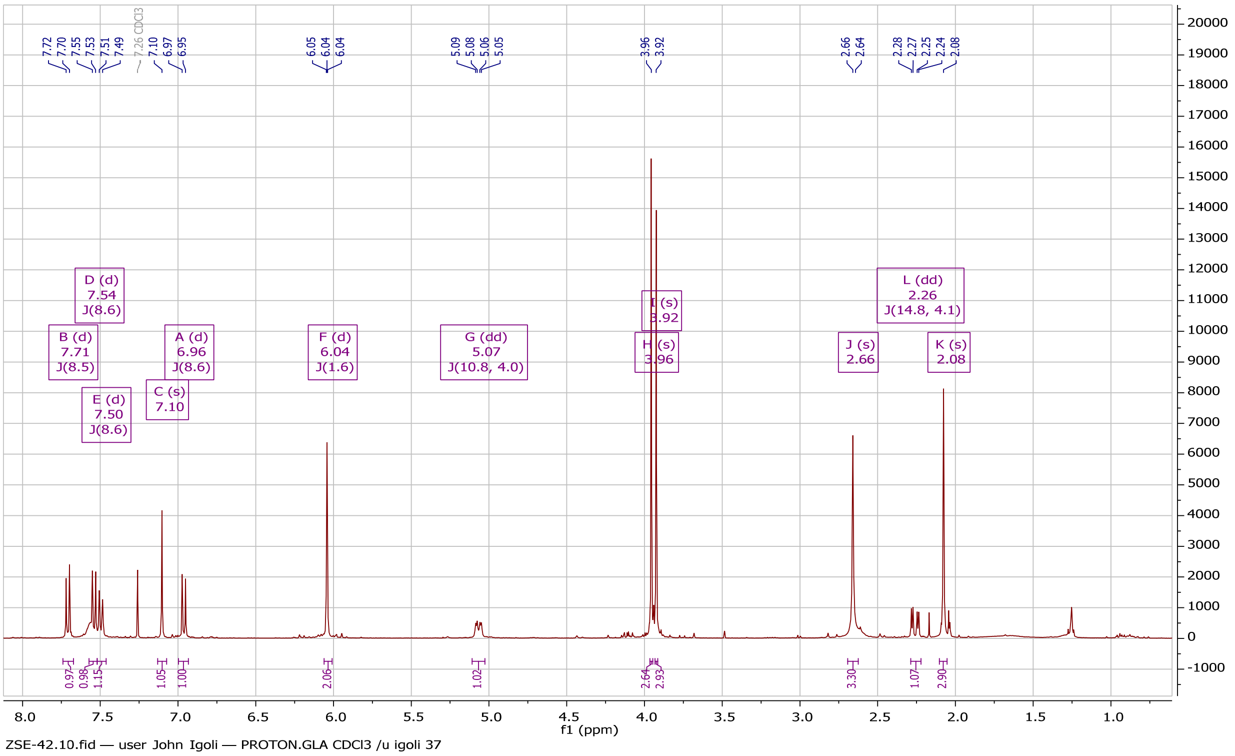

Figure S20: ^{1}H-NMR of dihydrochelerythrin **(6)**

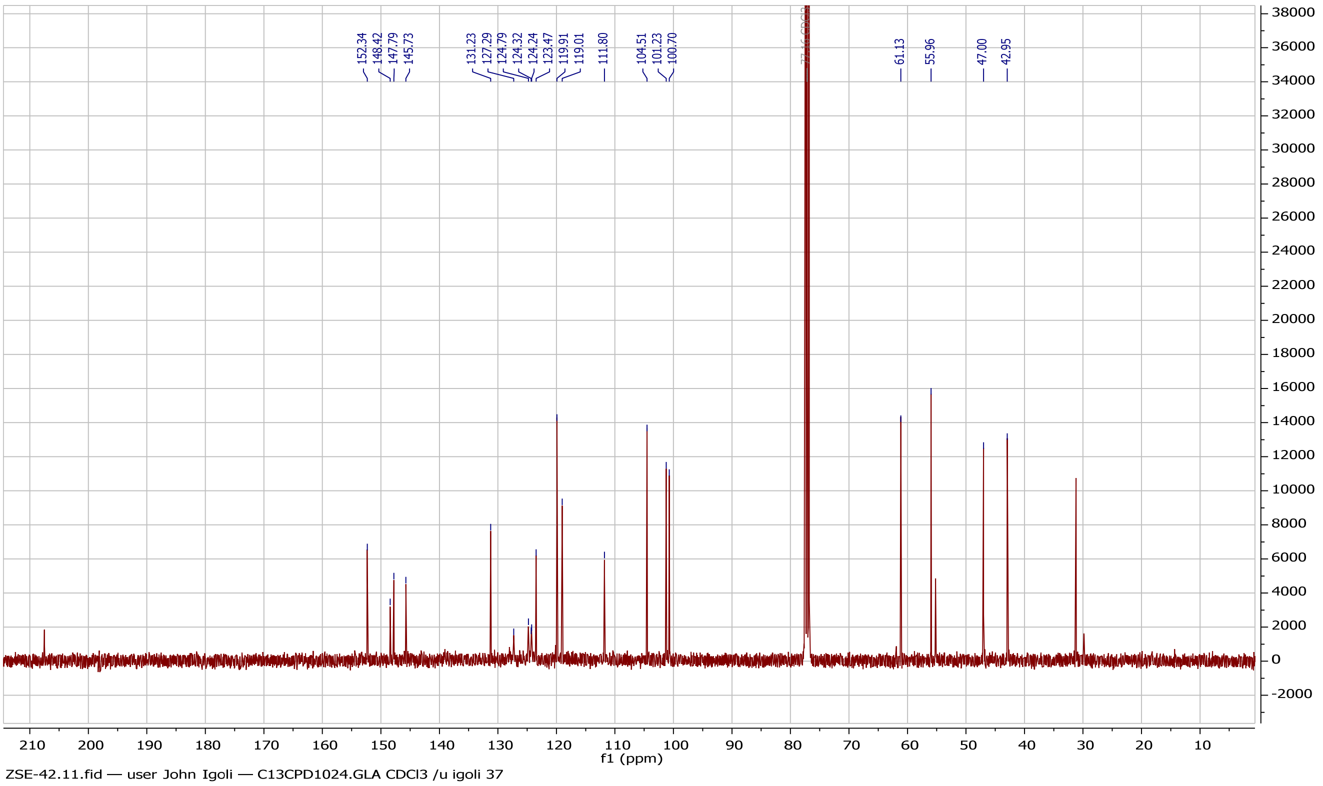

Figure S21: ^{13}C-NMR of dihydrochelerythrin **(6)**

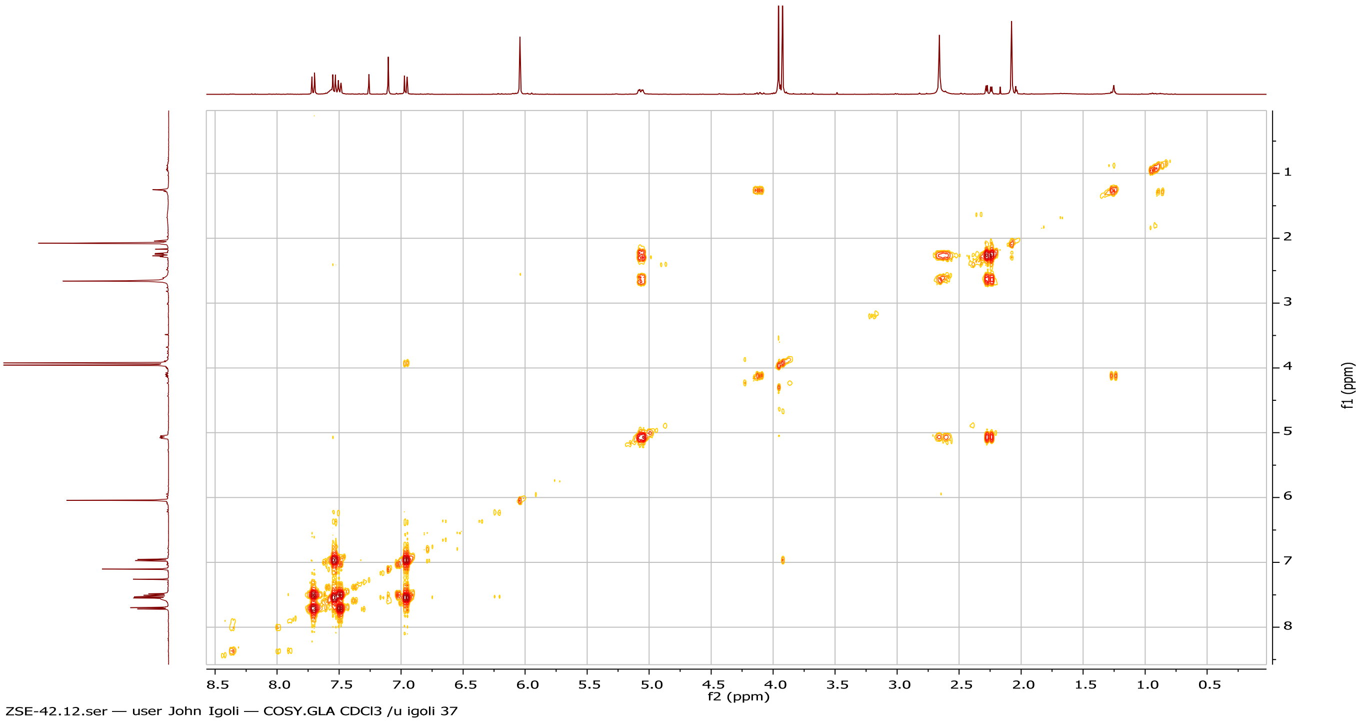

Figure S22A: COSY of dihydrochelerythrin **(6)**

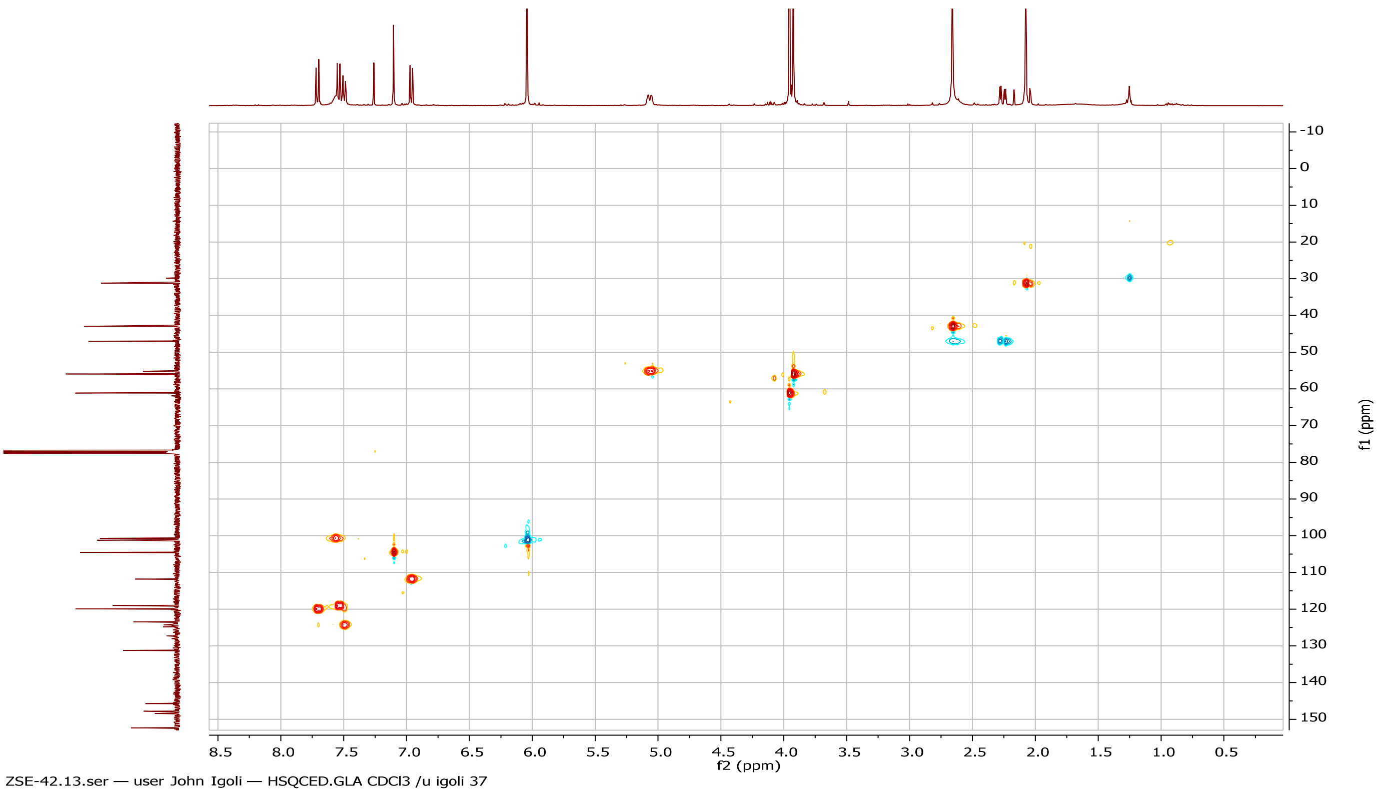

Figure S22B: HSQC of dihydrochelerythrin **(6)**

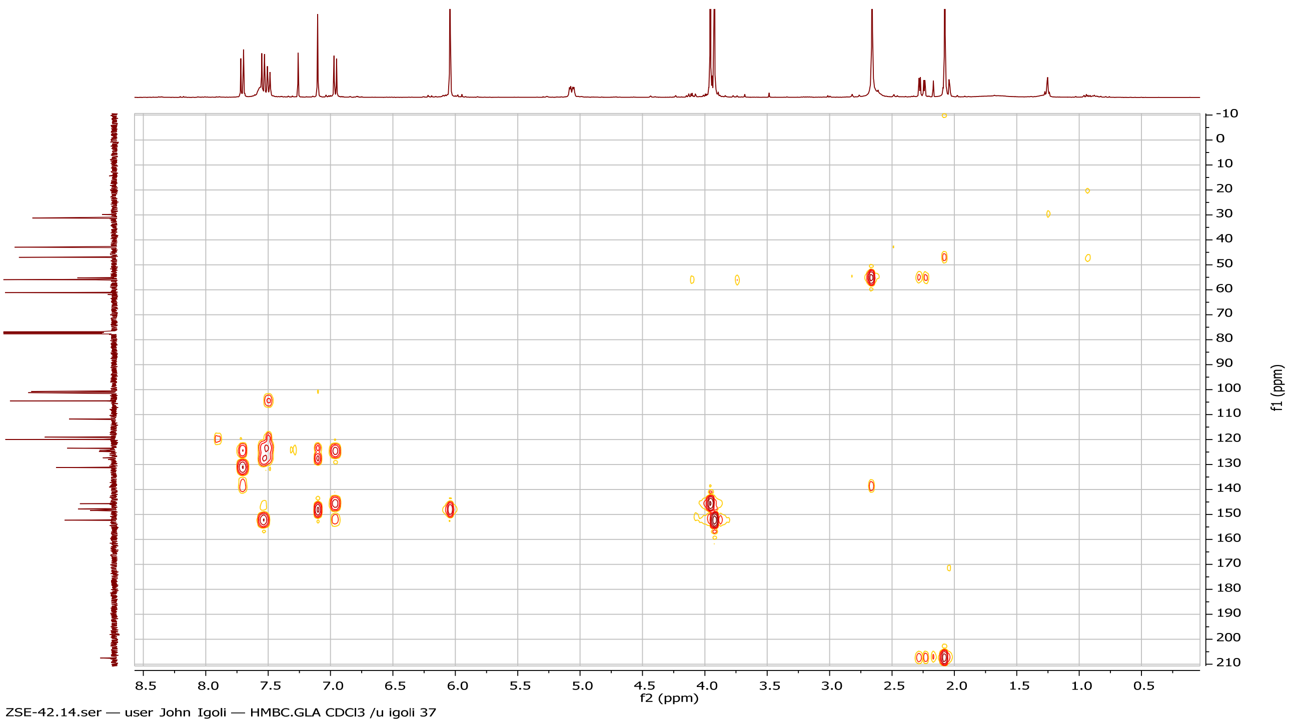

Figure S22C: HMBC of dihydrochelerythrin **(6)**

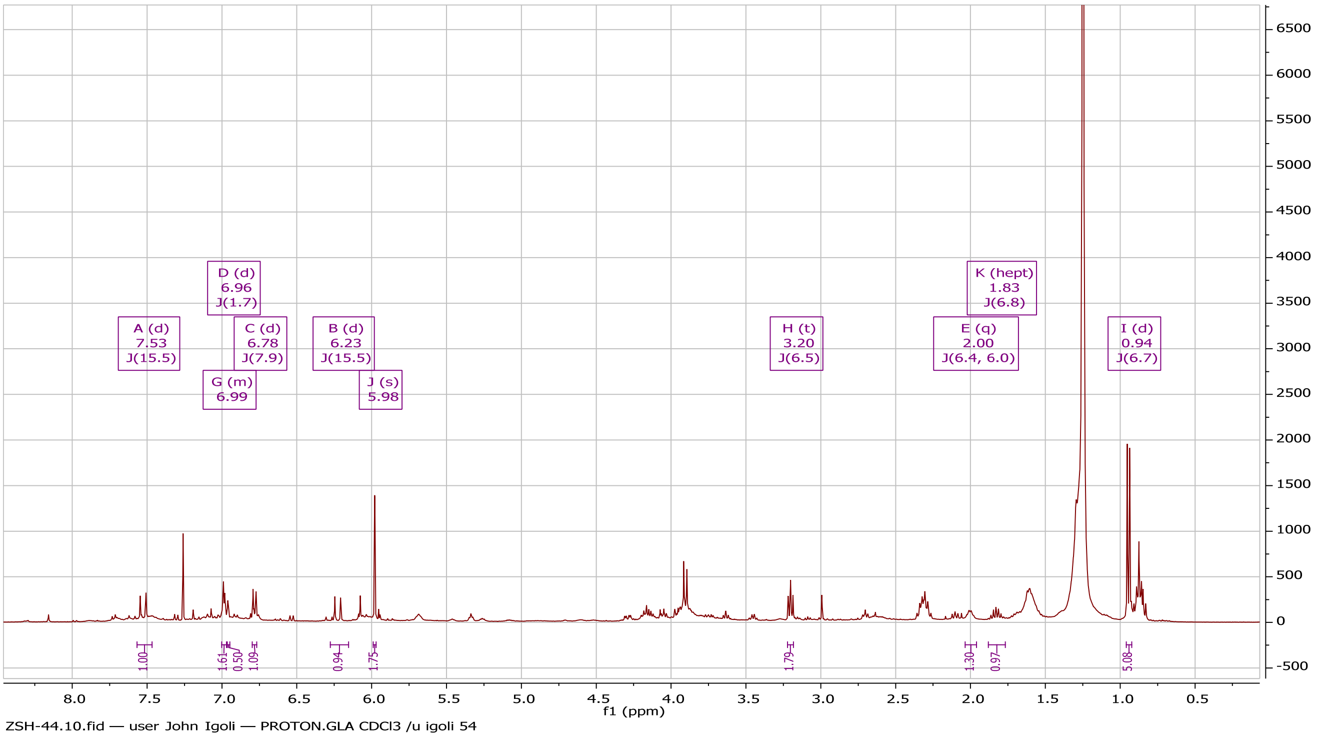

Figure S23: ^{1}H-NMR of fagaramide **(7)**

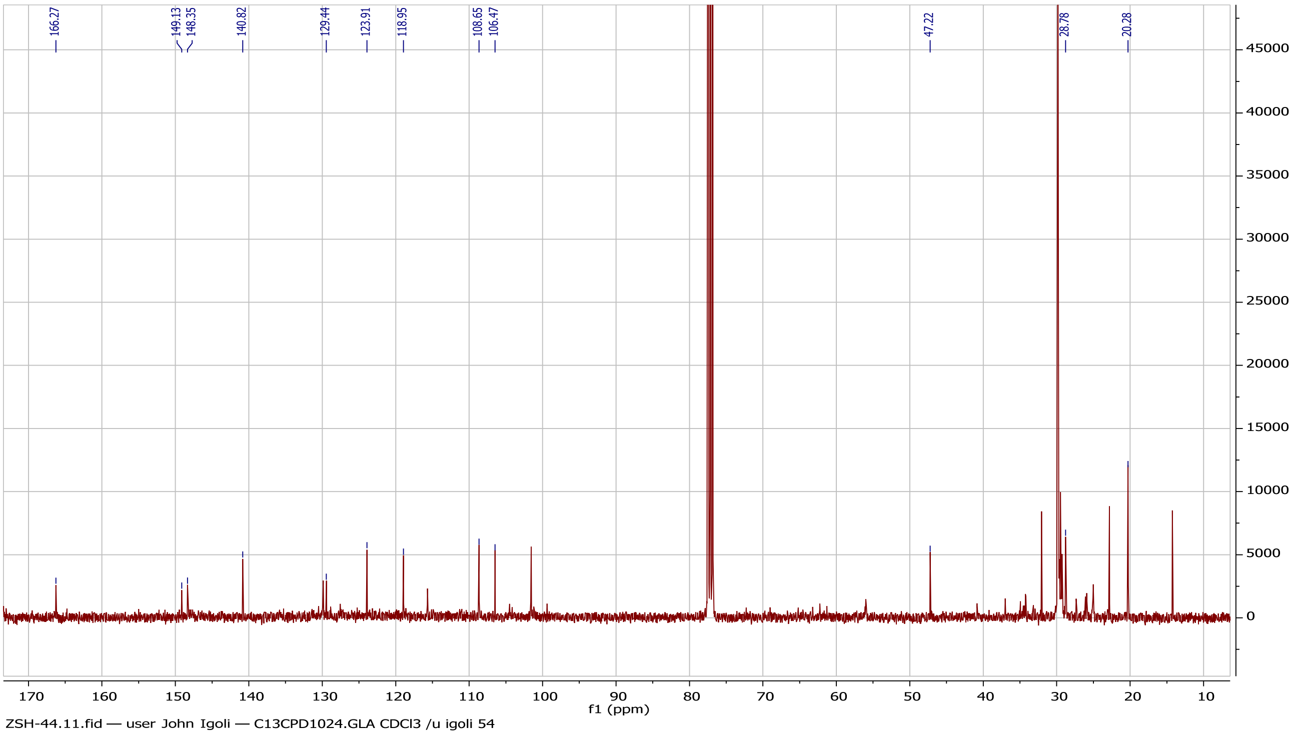

Figure S24: ^{13}C-NMR of fagaramide **(7)**

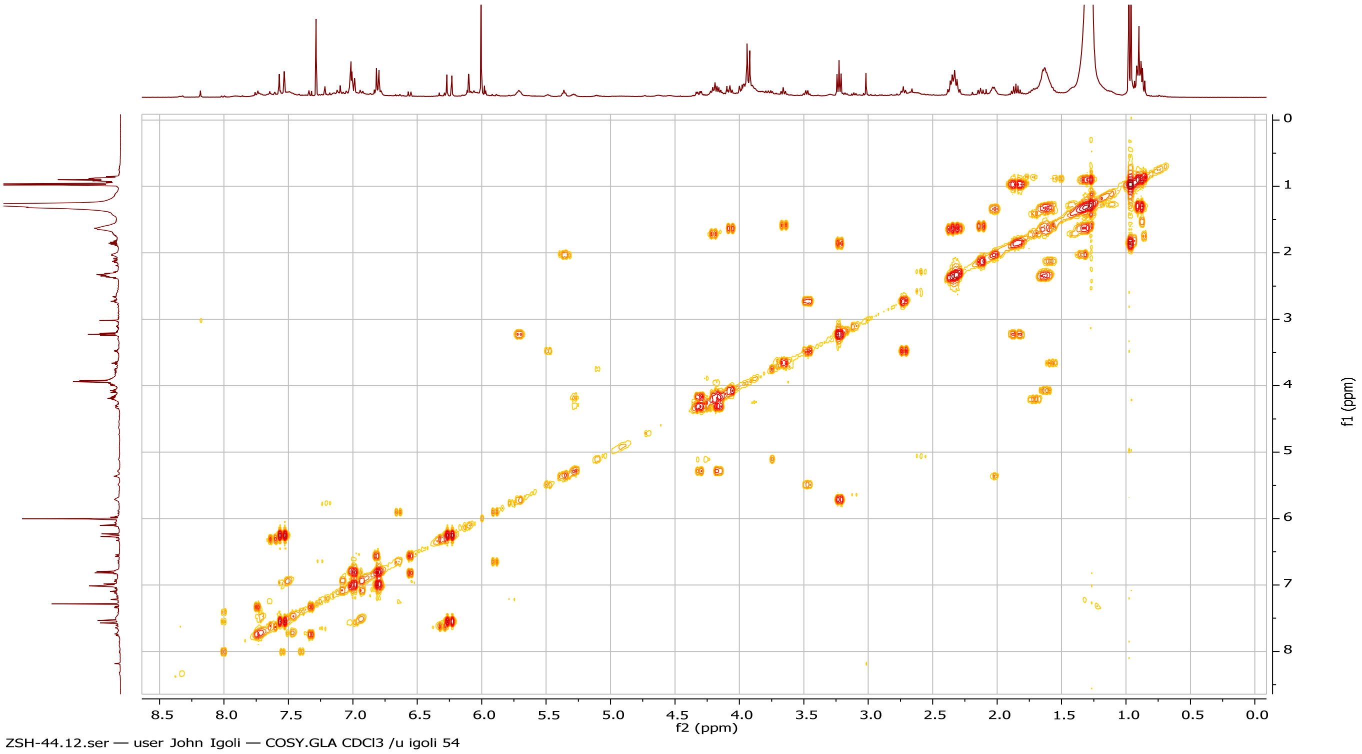

Figure S25A: COSY of fagaramide **(7)**

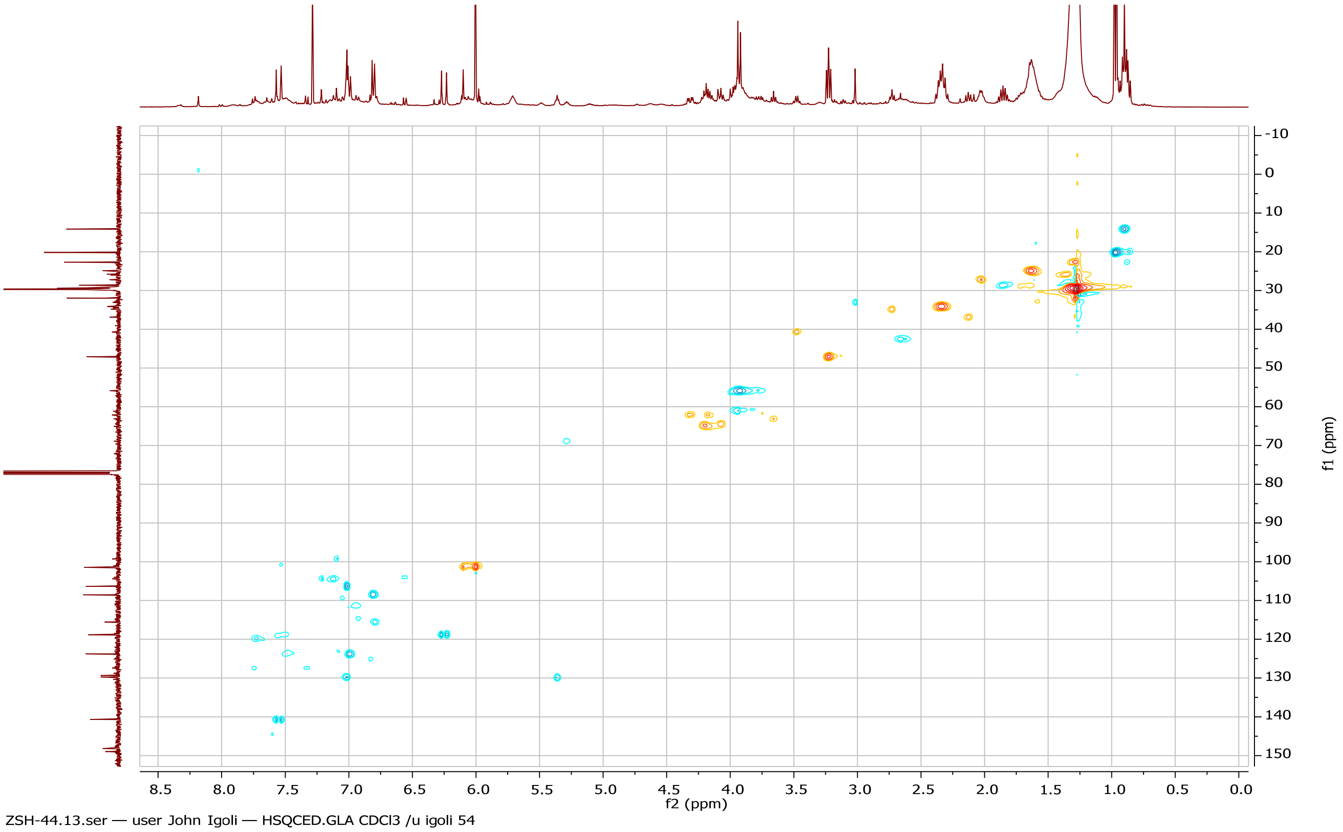

Figure S25B: HSQC of fagaramide **(7)**

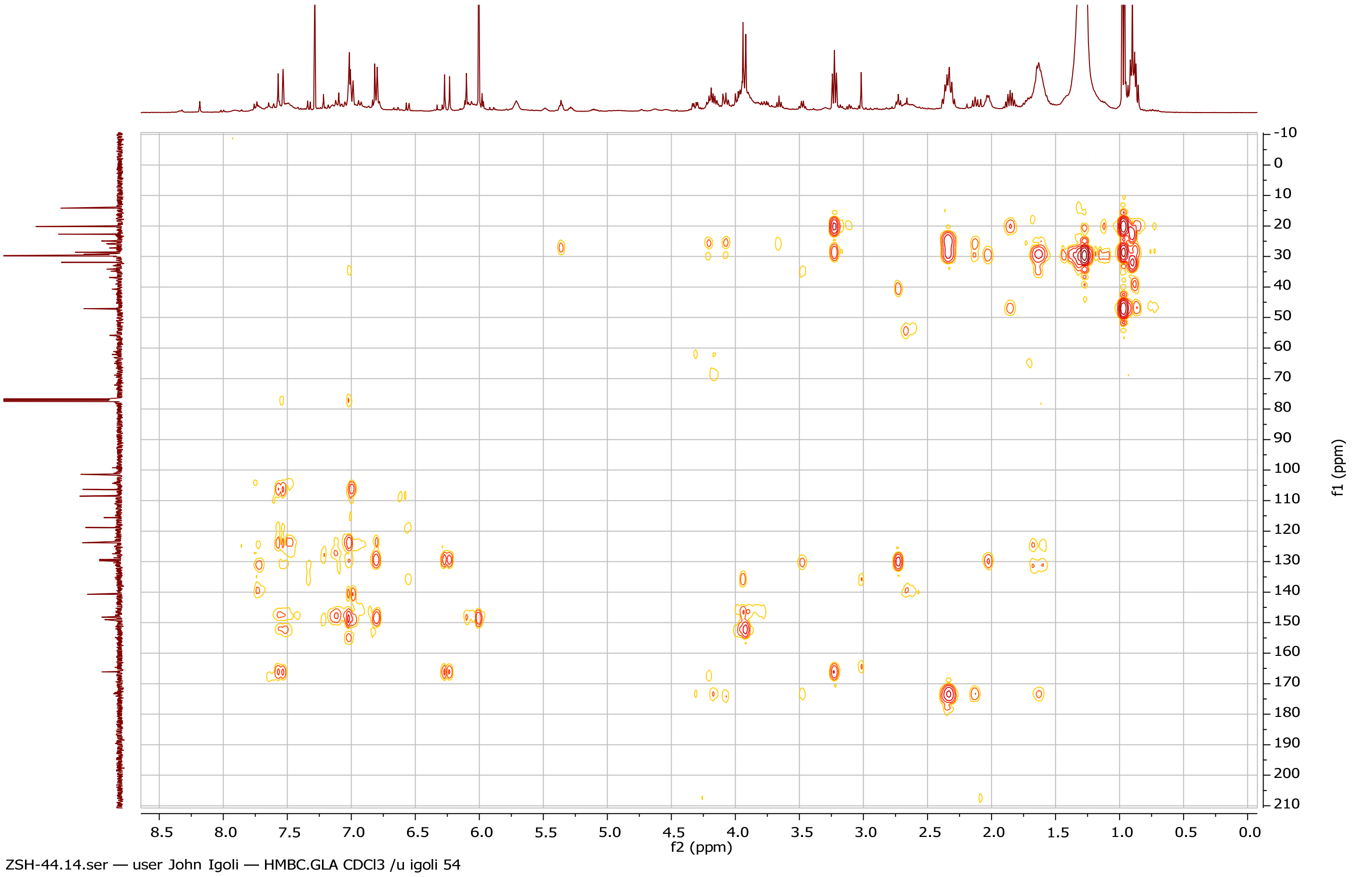

Figure S25C: HMBC of fagaramide **(7)**
